# Recycling of Polymerase Chain Reaction (PCR) Kits

**DOI:** 10.1021/acssuschemeng.2c07309

**Published:** 2023-03-24

**Authors:** Weina Liu, Yong Zhu, Francesco Stellacci

**Affiliations:** † Institute of Materials, École Polytechnique Fédérale de Lausanne, Station 12, Lausanne 1015, Switzerland; ‡ Institute of Bioengineering, École Polytechnique Fédérale de Lausanne, Station 12, Lausanne 1015, Switzerland

**Keywords:** recycling DNA, PCR kit, phosphorothioate, ribose sugar modification, nonhydrolyzable primer

## Abstract

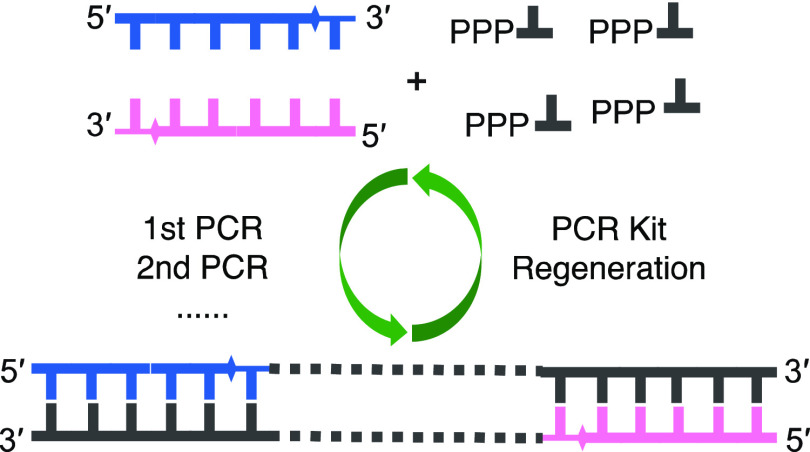

Polymerase chain reaction (PCR) kits have been used as common diagnosing tools during the outbreak of the severe acute respiratory syndrome coronavirus 2 (SARS-CoV-2) pandemic, with daily worldwide usage in the millions. It is well known that at the beginning of the pandemic, there was a shortage of PCR kits. So far, the ecosystem of a PCR kit is linear use; that is, kits are produced, used once, and disposed of as biolab waste. Here, we show that to mitigate the risk of future shortages, it is possible to envision recyclable PCR kits based on a more sustainable use of nucleic acid resources. A PCR kit is mainly composed of primers, nucleotides, and enzymes. In the case of a positive test, the free nucleotides are polymerized onto the primers to form longer DNA strands. Our approach depolymerizes such strands, keeping the primers and regenerating the nucleotides, i.e., returning the nucleic acid materials to the original state. The polymerized long DNA strands are hydrolyzed into nucleotide monophosphates that are then phosphorylated into triphosphates using a method that is developed from a recent publication. We used oligonucleotides with a 3′-terminal phosphorothioate (PS) backbone modification as nonhydrolyzable PCR primers, which are able to undergo the recycling process unchanged. The nuclease resistance of oligonucleotides with a ribose sugar modification was also evaluated, which showed worse recycling efficiency than PS-modified oligonucleotides. We successfully recycled both PCR primers and nucleotide monomers (∼75% yield). We demonstrate that the method allows for the direct reuse of PCR kits. We also show that the recycled primers can be isolated and then added to endpoint or quantitative PCR. This recycling approach provides a new path for circularly reusing nucleic acid materials in PCR kits.

## Abstract 2

The primers and nucleotides of a PCR kit could be recycled using nonhydrolyzable primers with a 3′-terminal phosphorothioate (PS) modification.

## Introduction

Since the worldwide outbreak of the severe acute respiratory syndrome coronavirus 2 (SARS-CoV-2) pandemic, massive polymerase chain reaction (PCR) testing has been required in many countries for diagnosing and screening.^1,2^ By the end of 2021, the worldwide cumulative covid test (PCR and antigen test) reached ∼4 billion.^2^ PCR testing kits are single-use and end up in the waste after testing, which is a linear “take–make–dispose” economy system.^3^ A large amount of biolab waste is then accumulated through this massive, daily repeated PCR test. Although many countries announced the endemic state of the SARS-CoV-2 pandemic in 2022, PCR and rapid tests are still provided due to the threat of reinfection and the emergence of new variants or other human infectious diseases (i.e., monkeypox).^4−6^ PCR is a polymerase-catalyzed, templated, temperature-controlled, precise DNA polymerization and amplification process. It seems necessary to consider the usage of nucleic acid materials in PCR kits in a more sustainable way by transitioning PCR testing kits from a linear economy to a circular economy.

**Scheme 1:**
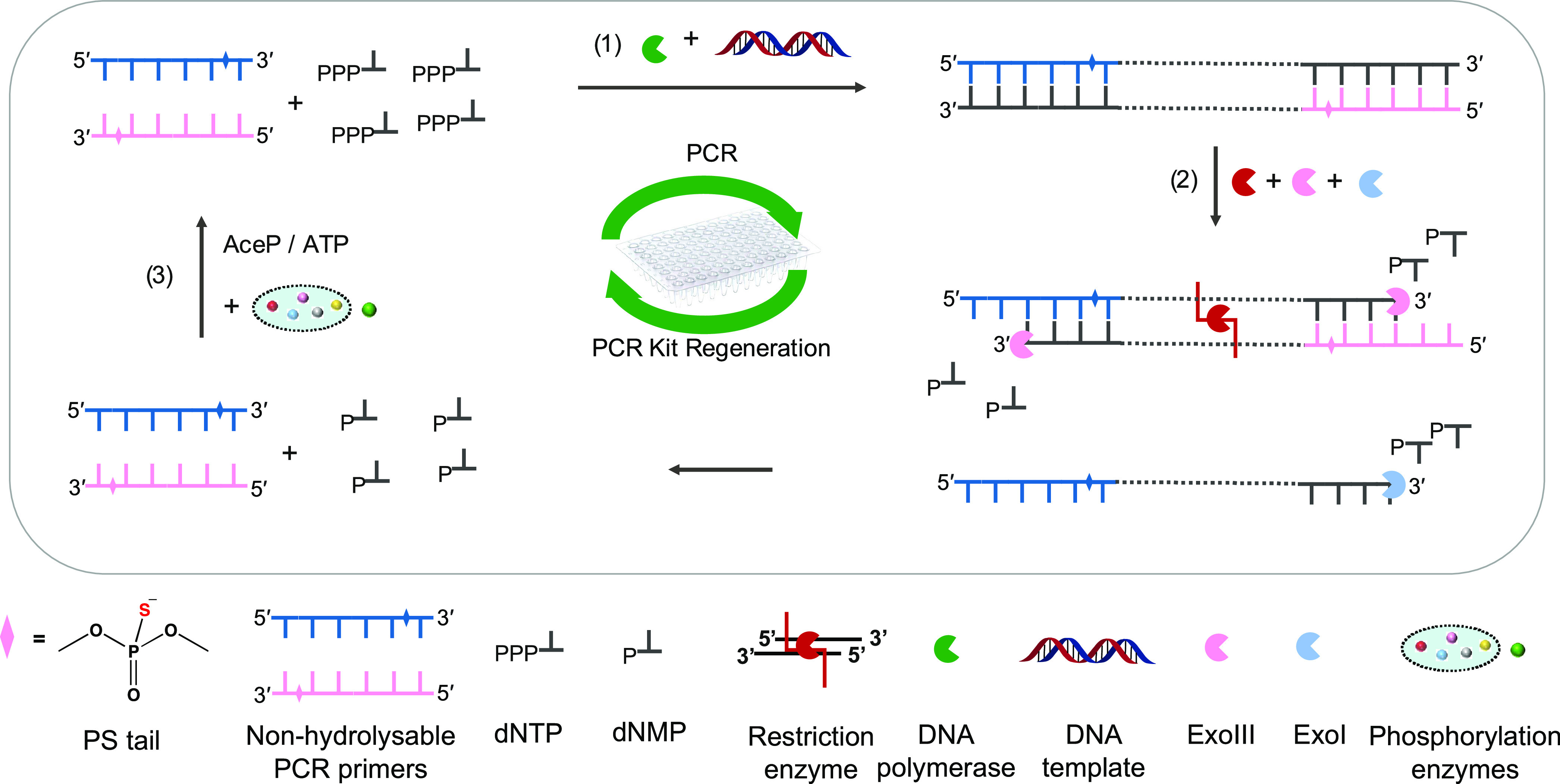
Three-Step Regeneration of a PCR Kit Using Nuclease-Tolerant Oligonucleotides with a Terminal Phosphorothioate (PS) Backbone Modification as Primers for PCR In the Legend, PS tail shows the chemical structure of phosphonothioate. The sulfur atom (red) is distinct from the oxygen atom in the naturally occurring phosphodiester of DNA. Step (1): PCR amplification using Primer-PS. The PS modification on primers does not affect the efficiency of PCR. Step (2): cleavage and hydrolysis of the PCR product catalyzed by restriction enzymes, Exo I and Exo lII. The primers with a PS modification can tolerate enzymatic hydrolysis in this step. Step (3): one-pot phosphorylation with enzymes and AceP/ATP to convert dNMPs into dNTPs.

A “circular economy” would change the economic logic by turning linear used old goods into new resources with the aim to form closed loops in the industrial ecosystem, minimize waste, and reduce the need to make the originals from scratch.^7^ For circular recycling of polymer materials (i.e., PCR waste), depolymerization is required to break them down into smaller units (often the starting oligomers or monomers), which, in turn, can be reused to produce new polymers or new molecules.^8,9^ In PCR kits, the initial nucleic acid materials are primers (oligonucleotides with a length of about 20 nucleotides) and nucleotide triphosphate monomers (dNTPs). The function of primers is sequential recognition and complementary binding to template DNA (e.g., the representative sequences of viral genomes). Under the catalysis of DNA polymerases, DNA chain elongation is processed (Scheme 1, step 1) by incorporating nucleotide monomers in the 5′-to-3′ direction, forming phosphodiester bonds as internucleotides linkages for DNA polymerization.^10^ In the case of a positive test, the PCR products (wastes) are the copies of the targeted DNA strand, which are polynucleotides conjugated by phosphodiester bonds. To circularly recycle the nucleic acid materials in PCR kits, a controlled depolymerization method is required, which should proceed in the 3′-to-5′ direction (the opposite direction of DNA polymerization). This reaction would break the phosphodiester bonds and release monomeric nucleotide monophosphates (dNMPs). Also, the depolymerization process should terminate at the 3′ terminus of PCR primers, leaving them intact to be reused.

Our group recently reported a recycling approach called a nature-inspired circular-recycling system (NaCRe) able to recycle natural sequence-defined polymers, such as proteins^11^ and DNA.^12^ Specifically, NaCRe can depolymerize DNA completely and recycle the nucleotide monomers into any new desired DNA sequence.^12^ NaCRe cannot be directly used for circular recycling of all nucleic acid materials from PCR kits. As described, it would hydrolyze (depolymerize) the entire chain of the DNA polymer, including the PCR primers. To circularly recycle the PCR primers, the depolymerization of the DNA polymer by the NaCRe system should terminate before the 3′ terminus of the primer, leaving the primer intact and ready to be reused (Scheme 1, step 2).

PCR primers are oligonucleotides synthesized by solid-phase chemistry. During the synthesis, chemical modifications of the nucleobases, sugar bonds, and internucleotide phosphodiester bonds can be readily introduced into the primers.^13,14^ To improve the nuclease resistance of primers and keep them intact for recycling, a 3′-terminal modification with the ability to resist hydrolyzation is required. In this paper, we replaced the natural phosphodiester with phosphorothioate (PS^15^) at the 3′ terminus of primers to obtain a nonhydrolyzable backbone. PS modification is more commonly used for developing a nuclease-resistant backbone^14,16,17^ for oligonucleotide drugs. The phosphodiester bonds of the nonbridging oxygen atoms were substituted with sulfur (in Scheme 1, see PS tail in the Legend), resulting in a slight lengthening of the P–S bond and enhanced resistance to nuclease degradation^18^ Hopefully, the PS-terminated primers can tolerate the enzymatic hydrolysis of exonucleases in the NaCRe system. Here, we could combine the DNA NaCRe system with primers that are terminally protected to achieve a system that, upon hydrolyzation, regenerates both the original primers and the nucleotides (Scheme 1). This DNA recycling approach brings a new perspective to the design of PCR kits.

## Results and Discussion

### PS-Modified DNA

To establish our method for recycling PCR kits, we first needed to determine whether the modified hydrolysis-resistant oligonucleotides could be used as primers in a PCR experiment. Forward and reverse primers were designed with a 4-PS tail (4 phosphorothioate linkages at the 3′ terminus, Scheme 1). The PS-modified oligonucleotides were used as PCR primers for the amplification of a luciferase DNA sequence (luc, amplification length 1653 base pairs^19^) as an arbitrary example of a biologically meaningful DNA sequence. Primers without the PS modification were applied for PCR as controls to compare the polymerization efficiency. The PCR amplification products were purified using a standard PCR extraction kit and quantified by Nanodrop.^20^ We found only a slight decrease in the PCR yield with PS-modified primers (Figure S1a), showing that the PS modification did not have a significant impact on the affinity of the primers to their DNA targets or the chain extension mediated by polymerase.

To test the hydrolysis resistance of backbone-modified primers, we used a one-pot DNA hydrolysis method that was developed recently.^12^ Primers labeled with a fluorescent dye at the 5′ terminus (with or without PS modification) were incubated together with nucleases to evaluate their nuclease tolerance. The reaction mixture was analyzed using agarose gel electrophoresis. We found that the PS-modified primers can tolerate more than 24 h of nuclease hydrolysis (Figure S1b), while the primers without PS modification were nearly completely hydrolyzed after 2 h. The PS-modified oligonucleotides showed excellent hydrolysis resistance, making them suitable for recycling.

### Recycling Nucleotide Monomers from a PCR Kit

**Figure 1:**
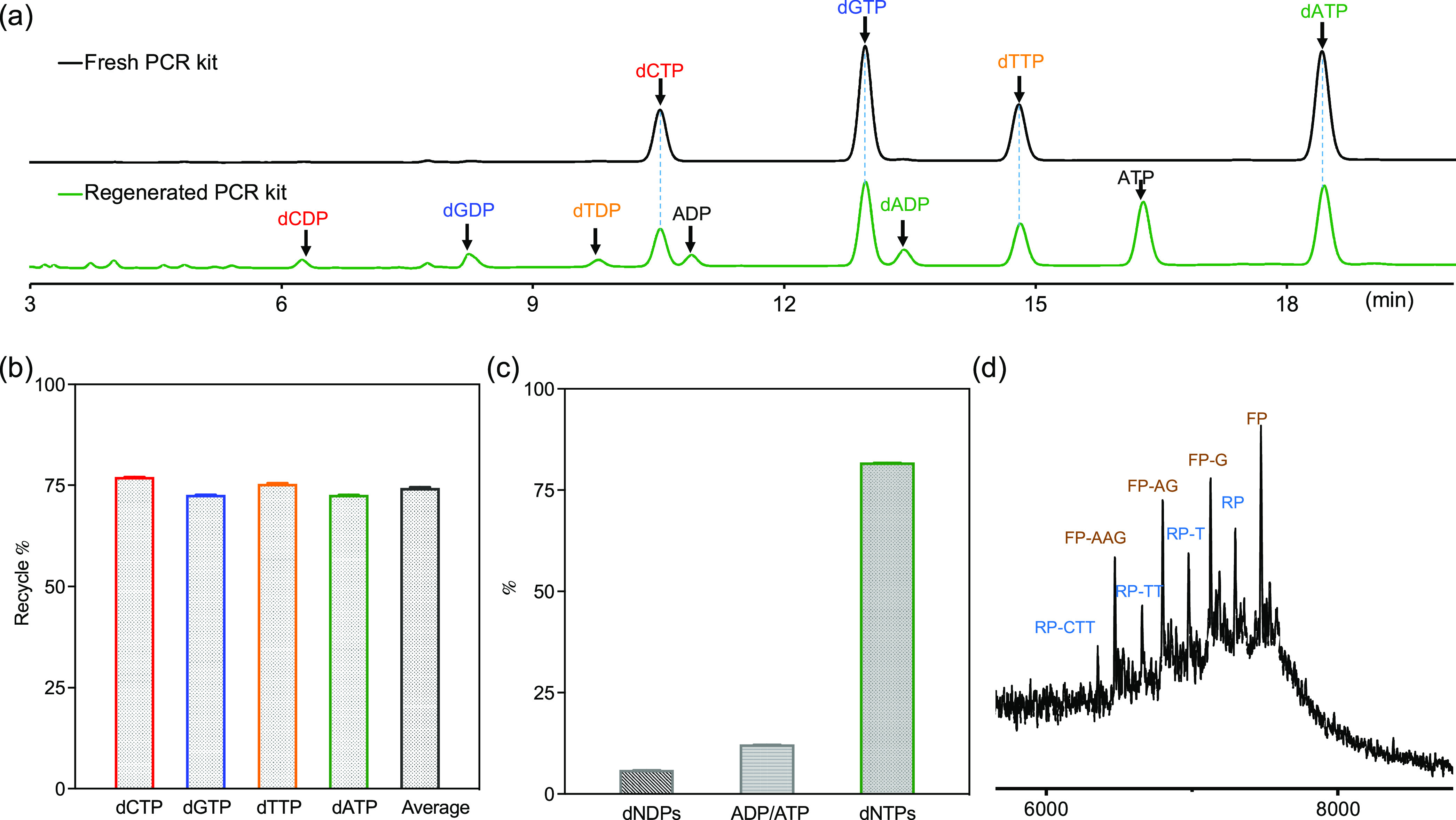
HPLC retention time of dNTP standards from a fresh PCR kit, and the products of the phosphorylation mixture from the regenerated PCR substrate. *X* axis: retention time; *Y* axis: absorption at 254 nm. (b) Recycling yield of dNTPs in the regenerated PCR substrate: dC 77.04 ± 0.05%, dG 72.64 ± 0.02%, dT 75.42 ± 0.19%, and dA 72.65 ± 0.04%; on average 74.44 ± 2.01%; for calibration curves of dNTPs and dNDPs, see Figure S2b,c. (c) Ratios of all nucleotide components in the regenerated PCR substrate: dNDPs 5.94 ± 0.04%, ADP/ATP 12.23 ± 0.02%, and dNTPs 81.81 ± 0.02%. (d) Mass spectra of the recycled forward and reverse primers with 0–3 mer terminal nucleotides after hydrolysis (FP lost terminal: G, AG, AAG; RP lost terminal: T, TT, CTT).

We then used such PS-modified primers in a PCR kit that led to successful DNA elongation (Scheme 1, step 1). The processes of consumption and recycling of nucleotide monomers by DNA polymerization and depolymerization were monitored by high-performance liquid chromatography (HPLC) (retention time plots in Figures 1a and S2a). In such plots, when comparing a fresh PCR kit with a used one (Figure S2a, lines 1 and 2), we find that about two-thirds of the dNTPs are consumed due to polymerization (for calculation details, see Methods), as expected. We also find new peaks of nucleotide diphosphates (dNDPs, Figure S2a, line 2). We believe that these dNDPs are derived from dNTPs due to their partial hydrolysis induced by heating under PCR conditions. Next, we applied the second NaCRe step to the PCR waste (see Scheme 1, step 2); that is, the overall mixture was hydrolyzed to obtain dNMPs. In this step, we modified the published protocol of hydrolysis to more efficiently hydrolyze all DNA templates and the PCR product. Two restriction enzymes (*Ban*I and *Bst*YI) with multiple cleavage sites of the amplified luc sequences were added to the PCR product so that the amplified DNA sequences can be fragmented into shorter pieces. Subsequently, the DNA fragments were hydrolyzed by a mixture of exonucleases to release dNMPs, as in the established method.^12^ After the enzymatic depolymerization process, the amount of dNMPs in the recycled PCR kit was highly increased (Figure S2a, line 3). Further, the released dNMPs were phosphorylated to generate dNTPs (Scheme 1, step 3), following the established one-pot phosphorylation method.^12^ In brief, the phosphorylation reaction was catalyzed by T4 NMP kinase and an *Escherichia coli* S30 extract (rich in phosphotransferase), with acetyl-phosphate (AceP)/ATP as the dual phosphate donor. After phosphorylation, the dNMP peaks almost disappeared, and the intensity of dNTP peaks increased (Figure S2a, line 4). Overall, through this hydrolysis–phosphorylation process, monomeric dNTPs in the PCR substrate were regenerated from PCR products/waste.

Concentrations of dNTPs from the fresh and regenerated PCR kits were determined (Figure 1a, with calibration curves of dNTPs in Figure S2b). After all of the steps of dNTP recycling, the reaction mixture was diluted 1.47 times due to the components added in the NaCRe steps (for calculation details, see Methods). The average concentration for each dNTP was decreased to ∼100 μM (Figure S2c). The average recycling efficiency of each nucleotide monomer was calculated to be 74.5 ± 2% (Figure 1b), which is relatively high, showing the feasibility of recycling monomeric nucleotide materials in PCR kits. The phosphorylation yield was relatively good (on average, 93 ± 2%; Figure S2d), which is similar to the phosphorylation yield of dNMPs reported by us.^12^ In the regenerated PCR kit, the dNDP residue was 5.94 ± 0.04%, the ATP/ADP residue was 12.23 ± 0.02%, and dNTP content was 81.81 ± 0.02% (Figure 1c). We notice that a fraction of the dNTPs was not fully hydrolyzed and retained as 3mer–6mer oligonucleotides in the recycled PCR kit (Figure S3).

### Recycling Primers from PCR Kits

We then evaluated whether the PS-modified primers can be preserved through this enzymatic hydrolysis and recycling process. After all three steps of NaCRe, we isolated the primers using a commercial oligo extraction kit. The molecular mass of the extracted primers was measured by matrix-assisted laser desorption/ionization-time-of-flight (MALDI-TOF) mass spectrometry. A mixture of recycled primers with a fully preserved sequence and shortened length (loss of 1, 2, or 3 terminal nucleotides) was observed (Figures 1d and S4a–c), showing that the 4-PS tail could effectively protect primers from the enzymatic hydrolysis step. Unexpectedly, we found that the enzymatic hydrolysis was “slowed down” instead of totally “stopped” by the 4-PS tail. For the preserved primers with 0–3 terminal nucleotides lost, the enzymatic hydrolysis was relatively slow, yet the remaining length of primers could be recycled. These observations lead us to believe that once all of the 4PS-modified nucleotides are hydrolyzed, the primers quickly degrade. A possible reason for the incomplete protection against hydrolysis could be the chiral configuration of phosphorothioate. Indeed, *S*-PS can tolerate enzymatic hydrolysis better than *R*-PS.^21^ As the 4PS-modified primers were achieved by solid-phase synthesis, it is difficult to determine the stereoselectivity of the PS modification at the current stage. Stereopure phosphorothioate-modified primers are reported,^22^ which could be more resistant to enzymatic hydrolysis. Overall, the recovery yields (for calculation details, see Methods) of primers were found to be 60.85 ± 0.01% (mass ratio) and 64.38 ± 0.01% (molar ratio, calculated from the average mass of recycled primers), which is rather promising. We should state that, due to the limited number of primers (∼0.7 μg), there was a significant loss of material in the oligo extraction process; hence, the yields we mention are lower estimates.

### Reused PCR Kit

**Figure 2:**
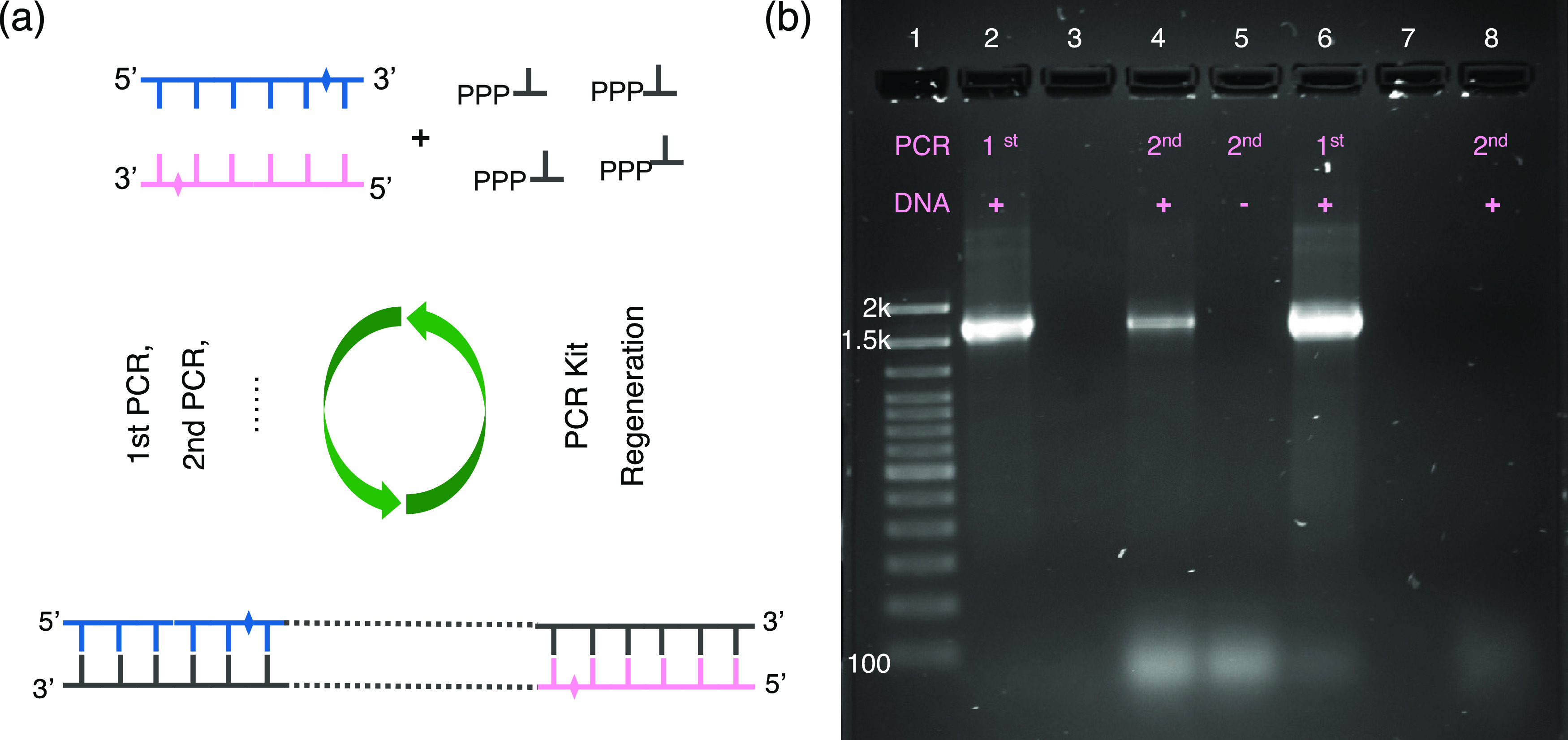
(a) Simplified scheme showing the circularly recycled and reused PCR kit. (b) Agarose gel electrophoresis of the PCR amplification product from fresh (1st) and regenerated (2nd) PCR kits: lane 1, ladder; lane 2, 1st PCR product using primer-PS; lane 3, hydrolyzed 1st PCR product; lane 4, 2nd PCR product with DNA template added; lane 5, 2nd PCR product without DNA template added; lane 6, 1st PCR product using primers without PS modification; lane 7, hydrolyzed 1st PCR product; lane 8, 2nd PCR product with DNA template added.

In an ideal situation, we would like to establish a method to regenerate and circularly reuse PCR kits by simply adding polymerization and depolymerization enzymes when needed (Figure 2a). Therefore, we tested the possibility of reusing the regenerated PCR substrate for a second round of PCR amplification. Only new DNA templates and polymerase were added to the regenerated PCR kit for the second round of PCR. The PCR amplification products (first and second, with or without adding the DNA template) and hydrolyzed PCR product were loaded onto agarose gel for analysis. Figure 2b shows the result of a positive PCR process, which has a clear band in the gel due to the elongation of the primers (lane 2). Lane 3 shows the hydrolyzed PCR material; the fact that the band of lane 2 cannot be seen reveals successful depolymerization of the PCR product. Lane 4 shows the result of a PCR process performed with a fully recycled kit. As expected, the band due to primer elongation can be seen again in this lane. The intensity of the band in lane 4 is weaker than that in lane 2. There are two reasons for this. First, there was a dilution when going from the original to the regenerated kit (overall 1.47× dilution), which led to a decreased DNA polymerization yield (lower intensity of the band in lane 4). Obviously, the second reason lies in the noncomplete recycling efficiency. However, it was still quite promisingly shown that the PCR reagents can be polymerized, depolymerized, and repolymerized in a closed loop. Without adding new DNA templates (Figure 2b, lane 5), the second PCR product was not obtained, showing that all of the added DNA template and the residue of the first PCR product were eliminated by enzymatic hydrolysis without causing contamination. The whole circulation of poly-oligo-/mononucleotides was also processed using primers without a PS tail protection as the negative control. The first (lane 6) and hydrolyzed PCR products (lane 7) were observed, but the second PCR product was not observed (lane 8), showing that without terminal PS protection, primers were hydrolyzed during the nucleotide recycling step. These results show that, using PS-modified primers, it is possible to circulate poly-oligo-/mononucleotide materials in PCR kits and reuse the PCR substrate in a closed loop. The system is still far from ideal. For example, we observed that in the recycled PCR kit, there is an additional band beside the expected band of PCR amplicons (∼100 bp, lanes 4 and 5). We believe that this band is probably due to the cleaved PCR amplicons with shorter lengths (Figure S3). In support of this conclusion, we should mention that when the recycled primers were purified using an Oligo extraction kit and applied for another round of PCR amplification, the additional band was absent (Figure S5).

### Recycled Primers for qPCR

We show that the nucleic acid materials in the PCR kit can be recycled using PS-modified primers. However, for the detection of specific DNA, an additional step of gel electrophoresis is required to show the band of the amplicon. This endpoint PCR is time-consuming and lacks efficiency. For molecular diagnostics, quantitative PCR (qPCR) is widely used as DNA amplification can be monitored in real time by measuring the fluorescence signal of the DNA intercalation dye. Primers are critical components in qPCR, as their functions of recognizing and binding with target DNA determine the precision and sensitivity of the molecular diagnostic assay.^23^ Consequently, in this circular recycling process, the quality of recycled primers (purity and sequence length) would directly affect the performance of qPCR. Earlier, we have shown that the 4PS modification can protect primers from enzymatic hydrolysis, but the recycled primers have different lengths (with 0–3 nucleotide loss); this, in theory, should lead to the use of a more complex annealing temperature profile for optimized recycled PCR use.

To maintain the length of the recycled primers without changing their annealing temperature significantly, primers with a 2-PS tail at the 3′-terminal modification were used for qPCR (amplicon length 133 nucleotides) and recycled by the abovementioned method. The qPCR performance was not affected by primers with a 2PS modification (Figure S6a). The 2PS-modified primers also showed excellent nuclease hydrolysis tolerance (Figure S6b). Further, the qPCR product was incubated with a mixture of nucleases for different periods (1, 2, and 4 h) to hydrolyze the amplicon and recycle the primers. The recycled primers were then purified using a commercial oligo extraction kit. Next, the Agilent Oligo Pro II system was applied to analyze the recycling efficiency of the 2PS-modified qPCR primers. As a capillary electrophoresis analytical tool, the Oligo Pro II system uses a denaturing gel that allows single-stranded oligonucleotide samples to migrate through a capillary and separate by size, allowing the direct detection of oligos with single-nucleotide resolution.

**Figure 3:**
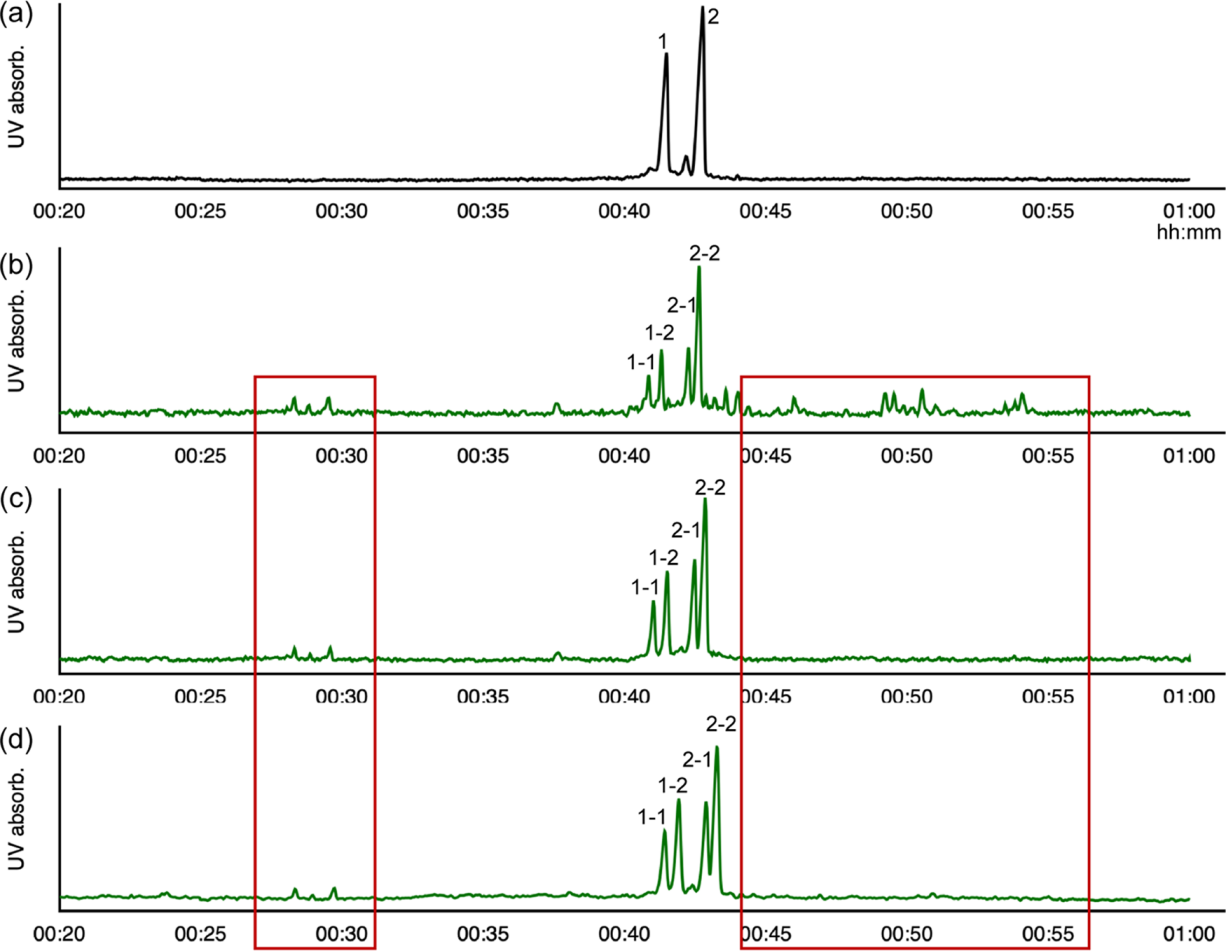
(a) Characterization of qPCR primers by Oligo Pro II capillary electrophoresis. Retention times (20–60 min) of fresh forward primers (peak 1) and reverse primers (peak 2) with a 3′ terminal 2PS modification. (b–d) Monitoring of the hydrolysis process of the qPCR product as well as the recycling process of primers by Oligo Pro II capillary electrophoresis. The retention time of recycled primers from the qPCR kit after (b) 1, (c) 2, and (d) 4 h of hydrolysis by a mixture of nucleases; the primers were purified using an Oligo extraction kit before injection into the Oligo Pro II capillary electrophoresis system. The areas enclosed in a red box in (b–d) represent the unhydrolyzed shorter oligonucleotide residues (left area) and longer qPCR amplicon residues (right area).

**Figure 4:**
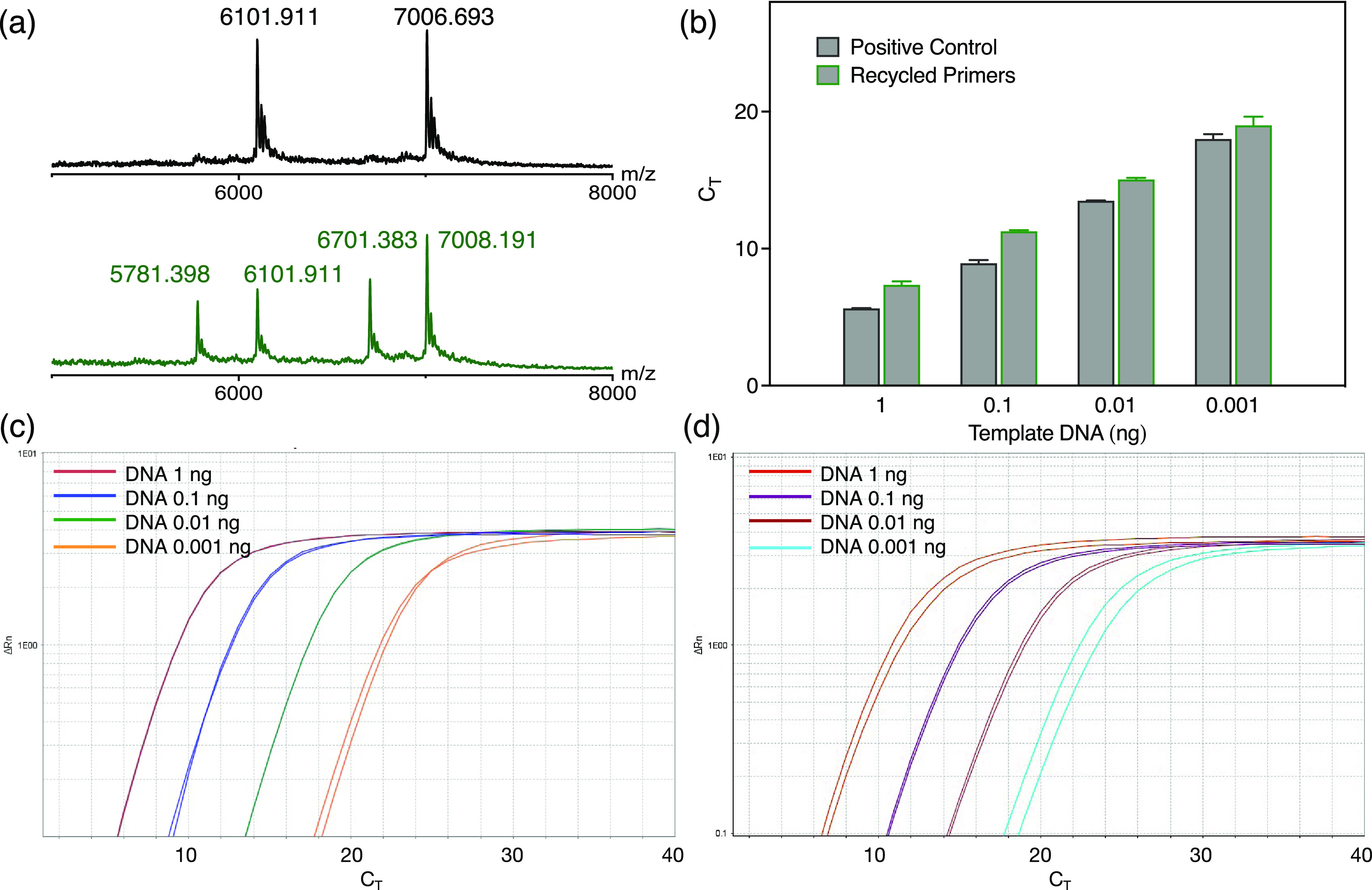
(a) MALDI-TOF characterization of fresh and recycled primers with a 2-PS tail modification. (b) CT values of qPCR amplification with fresh and recycled primers; DNA templates were 1, 0.1, 0.01, and 0.001 ng, respectively. qPCR amplification plots with (c) fresh and (d) recycled primers.

As shown in Figure 3a, for fresh forward and reverse primers (20 and 23 nt, respectively), the migration times were different and thus can be separated well (peaks 1 and 2, migration times between 40 and 45 min). The difference in the peak intensity (ultraviolet (UV) absorbance at 260 nm) between the forward and reverse primers is due to differences in their lengths and sequences (i.e., base content). As shown in Figure 3b, for the recycled primers, after 1 h of incubation with nucleases, there are four major peaks observed from the primer recycling products. They include recycled primers with fully preserved lengths and reproducible migration times similar to those of fresh primers (peak 1-2 and 2-2) and recycled primers with one additional terminal nucleotide hydrolyzed, showing shortened migration times (peak 1-1 and 2-1). These results show that the 2PS modification can effectively protect the primers from nuclease hydrolysis. The recycled primers can be either fully preserved or lose 1 nt. Further, the mixture of recycled primers was characterized by MALDI-TOF. Similar to the Oligo Pro II characterization, fully preserved primers and primers with only one terminal nucleotide lost were obtained (mass spectra in Figure 4a).

In addition, there were still several longer oligonucleotides observed after 1 h of nuclease hydrolysis. In the plot, these peaks occur between 45 and 55 min, whose migration time is longer than those of the fresh or recycled primers (Figure 3b, enclosed in a red box). These longer oligonucleotides should be residues of qPCR amplicons. After a longer incubation time (2 and 4 h), the longer oligonucleotide residues were not detected anymore (Figure 3c,d), showing that the qPCR residues can be fully eliminated by nuclease hydrolysis. In Figure 3b, one can also observe a few very short oligonucleotide residues (migration times between 25 and 30 min, enclosed in a red box) due to incomplete hydrolysis. The amount of very short oligonucleotide residues also decreased with prolonged hydrolysis time (Figure 3c,d).

### Reused qPCR Primers

The recycled primers were applied for qPCR amplification, with fresh primers used as positive controls (Figure 4). Similar cycle threshold (C_T_) values were obtained for the qPCR assay prepared with fresh primers and recycled primers (Figure 4b–d), showing that the recycled primers can be circularly used as new materials for qPCR amplification and DNA testing. The recovery yield of qPCR primers is 40.9 ± 0.5% (for calculation details, see Methods). Because of the loss of primers during oligo extraction, the recovery yield is a lower estimate of the recycling yield of the PS-modified primers. Even with only 2-PS modification, the qPCR primers can be effectively preserved and circularly reused in qPCR assays without a marked decrease in the detection sensitivity (less than 2 thermocycles). For the no-template control (NTC) sample, the C_T_ value of 22 is lower than that of the control sample (32 thermocycles, Figure S7b), indicating the presence of unwanted residues. Although this NTC result should not affect the detection limit of the qPCR kit, because it is higher than the C_T_ value of 0.001 ng found for the DNA template with the recycled primers (20, see Figure 4b), it would be better to purify the recycled primers on a larger scale (for example, by ion-exchange chromatography) to eliminate residues of DNA amplicons as well as any other potential contaminants.

### qPCR Primers with Ribose Sugar Modification

**Figure 5:**
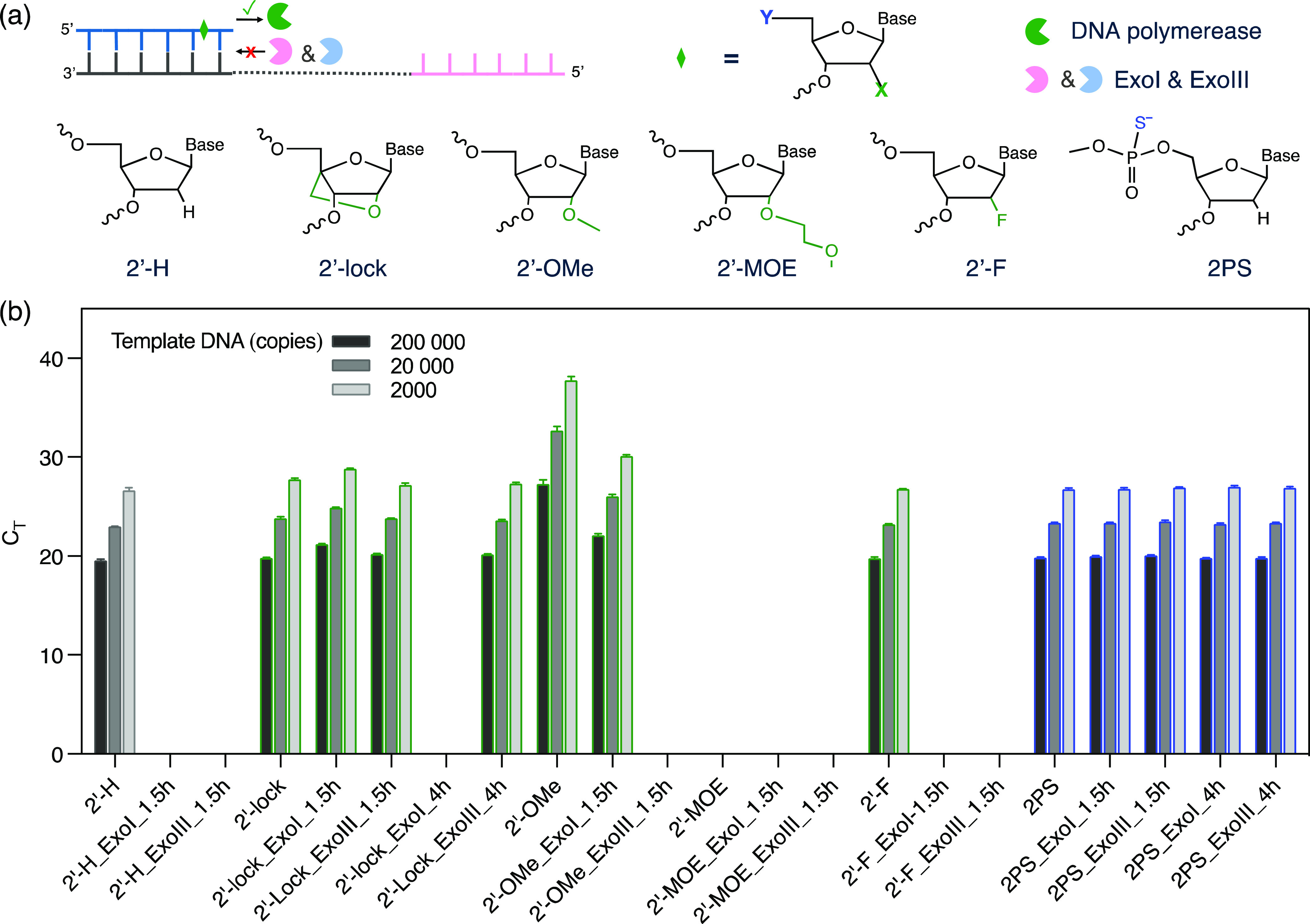
(a) Ribose sugar or backbone modification of qPCR primers (labeled as X or Y). X represents 2′-H (no modification), 2′-Lock (2′-locked nucleic acid), 2′-OMe (2′-*O*-methyl), 2′-MOE (2′-*O*-methoxyethyl), or 2′-F (2′-deoxy-2′-fluoro); Y represents the phosphodiester bond backbone modification of qPCR primers. (b) C_T_ values of qPCR amplification with chemistry modified primers. These primers were either without enzyme treatment, or with 1.5 and 4 h of nuclease Exo I or Exo III treatment. The amounts of DNA template added for qPCR were 200,000, 20,000, and 2000 copies, respectively. All of the nontemplate control (NTC) samples have C_T_ values that are either higher than 35 or undetermined.

In addition to the PS modification (phosphodiester backbone), ribose sugar modification can also improve the nuclease tolerance of oligonucleotides by increasing steric hindrance.^24^ For example, a 2′-Lock modification of oligonucleotides is formed by a bicyclic furanose unit that covalently conjugates the second and fourth carbons of the ribose sugar ring, mimicking sugar conformation.^25^ Oligonucleotides with one or several 2′-Lock modifications have shown excellent binding affinity to their complimentary strands.^25^ Also, the 2′-Lock modification at the penultimate (L-2, the last but one nucleotide) position generates excellent nuclease resistance.^26^ We tested the nuclease resistance of four types of ribose sugar-modified primers (Figure 5a) in terms of nuclease hydrolysis tolerance as well as DNA polymerization efficiency. Primers with 2′-Lock, 2′-OMe, 2′-MOE, 2′-F, and 2PS modifications and unmodified primers were incubated with nuclease Exo I and Exo III for 1.5 or 4 h. Next, the reaction mixtures were heated to 80 °C and incubated for 15 min to deactivate the nucleases. The six groups of primers were applied in qPCR for the amplification of the plasmid DNA template encoding the SARS-CoV-2 envelope sequence (2019-nCoV_E Positive). As shown in Figure 5b, primers with 2′-Lock, 2′-F, and 2PS modifications showed C_T_ values equivalent to that of the unmodified primers (2′-H), while the C_T_ value of 2′-OMe primers was increased. The 2′-MOE modification led to no detectable amplification. These results show that 2′-Lock, 2′-F, and 2PS are the most suitable modifications for qPCR primers as they showed a PCR efficiency similar to that of unmodified primers. After nuclease treatment, primers with a 2′-Lock modification could tolerate Exo I treatment for 1.5 h with a slightly increased C_T_ value and Exo III treatment for 4 h without any obvious change in the C_T_ value. They cannot tolerate 4 h of Exo I treatment as the C_T_ value was beyond the detection limit, which corresponds to the MALDI-TOF result (Figure S8a). Primers with a 2′-F modification could not tolerate the hydrolysis by both Exo I and Exo III for 1.5 h. In conclusion, primers with the 2PS modification showed the best nuclease resistance. After 1.5 and 4 h of Exo I and Exo III treatment, respectively, there were no obvious changes in the C_T_ values for qPCR, indicating that the concentration of primers was not drastically decreased by nuclease resistance. Although it is difficult to determine the recycling yield, qPCR results showed relatively good nuclease tolerance of the 2PS-modified primers. Both the intact primers and primers with one terminal nucleotide lost were detected by MALDI-TOF (Figure S8c,d), showing that the 2PS modification can effectively protect the primers from nucleases hydrolysis. After nuclease treatment, the concentration of 2PS-modified primers should remain the same, given that the qPCR sensitivity remains unchanged.

**Figure 6:**
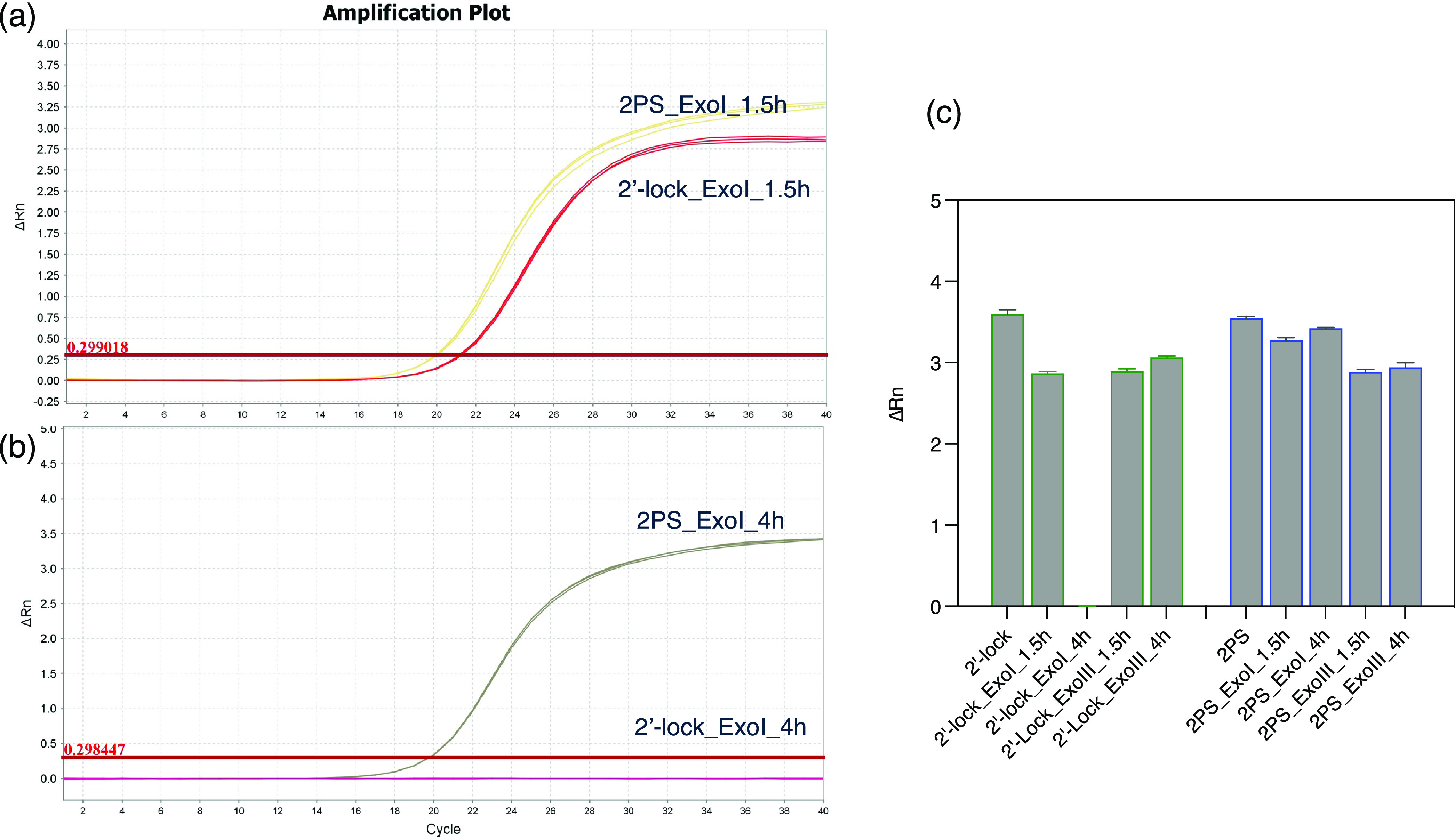
(a, b) qPCR amplification curves and (c) final ΔRn value of primers with 2′-Lock and 2PS modifications after nuclease treatment.

ΔRn indicates the magnitude of the signal (intercalation dye of SYBR Green) generated under the given set of PCR conditions.^27^ The final ΔRn value is correlated to the DNA synthesis yield of the PCR reaction, which is determined by the concentration of reactive agents in the PCR reaction (In this work, the ΔRn value is determined by the concentration of primers that can tolerate nuclease hydrolysis). As shown in Figure 6a,b, under the same nuclease treatment conditions, a higher final ΔRn value of qPCR was obtained for primers with a 2PS modification than those with a 2′-Lock modification. These results showed that 2PS can provide better protection than 2′-Lock for primers to resist nuclease hydrolysis. Also, for primers with a 2PS modification, with prolonged Exo I and Exo III incubation times (Figure 6c), there is no decrease in the final qPCR ΔRn value, showing that the concentration of primers was not significantly decreased by prolonged nuclease hydrolysis. Overall, the 2PS modification of primers slows down the nuclease hydrolysis effectively, making the circular recycling of qPCR primers possible.

## Conclusions

In this work, we discussed the possibility to circularly recycle the nucleic acid materials in PCR kits. Using PS modifications, the primers can be protected from the nucleotide recycling process, preserved, and extracted for circular usage. Together with the recycled monomeric nucleotides with good yield, the PCR substrate was regenerated and reused for the detection of targeted DNA. The PS-modified primers can also be recycled separately by the same method and reused as new materials for targeted DNA detection by qPCR with relatively good sensitivity. The quality (length and purity) of recycled primers can be evaluated by Oligo pro II capillary electrophoresis with single-nucleotide resolution. The method we established here shows the possibility of using PCR reagents in a smarter way. With the PS modification, it is possible to design nonhydrolyzable, recyclable primers to ease the problem of PCR kit shortage. It is possible to improve the recycling efficiency of primers using stereopure PS modifications in the future.^22^ Yet, large-scale recycling by this method will require the optimization of all steps and will not be easy. Overall, the daily accumulated PCR waste during the current pandemic could become a valuable resource for the circular recycling of PCR reagents.

So far, the established method was tested by endpoint PCR and qPCR (with intercalation dye), but it might also be applicable to recycle oligo- and mononucleotides from other DNA polymerization systems such as the loop-mediated isothermal amplification (LAMP) system.^28^ As DNA technology has become a very well-established technique with DNA materials used for sequencing, bioengineering, molecular diagnoses, DNA origami, DNA hydrogel, and DNA for data storage,^29−35^ our established DNA recycling methodology could also be adapted for the circular recycling of modified nucleotides and oligonucleotide products from the abovementioned waste to render more sustainable applications.

## Methods

### Materials

#### Chemicals and Consumables

Ethanol, isoproponal, and 2-mercaptoethanol (for oligo-extraction) were purchased from Sigma-Aldrich. 2′-Deoxyguanosine 5′-monophosphate sodium salt hydrate (dGMP), thymidine 5′-monophosphate disodium salt hydrate (dTMP), 2′-deoxycytidine 5′-monophosphate sodium salt (dCMP), 2′-deoxyadenosine 5′-monophosphate (dAMP), acetylephosphate lithium potassium salt (AceP), sodium chloride (DNase-, RNase-, and protease-free), sodium hydroxide solution (5.0 M), acetic acid, tetrabutylammonium dihydrogenphosphate solution (1.0 M in water), deoxynucleotide mix reagent (dNTPs, 10 mM each), and adenosine 5′-triphospate disodium (ATP, 100 mM) were purchased from Sigma-Aldrich. Nuclease-free water (10 × 50 mL) was purchased from Qiagen.

#### Self-Made Phosphorylation Buffer

HEPES buffer (1 M) (HEPES, 55 mM;, magnesium acetate, 15 mM; pH 7.5) was purchased from Thermo Fisher Scientific.

##### Chemicals for Gel Electrophoresis

For gel electrophoresis, 400 μL SYBR Safe DNA Gel Stain, DNA loading dye, SDS solution (6×), TAE buffer (Tris–acetate–EDTA) (50×), TrackIt 100 bp DNA Ladder, and Ultra Low Range DNA Ladder were purchased from Thermo Fisher Scientific. Agarose was purchased from Bio-Rad Laboratories. NuPAGE 4–12%, Bis–Tris, 1.0 mm, and Mini Protein Gel (12-well) were purchased from Invitrogen.

##### PCR and qPCR Reagents

DreamTaq Green PCR Master Mix (for the first round of PCR), DreamTaq polymerase (for the second round of PCR), and PowerTrack SYBR Green Master Mix (for qPCR) were purchased from Thermo Fisher Scientific. Amicon Ultra 0.5 mL Centrifugal Filters (3 kD cutoff) were purchased from Merck. The Oligo Clean-Up and Concentration Kit was purchased from Norgen Biotek.

##### Enzymes

Restriction enzymes HpyF3I (*Dde*I), BshNI (*Ban*I), PsuI (BstYI), and Kpn2I (*Bsp*EI) were purchased from Thermo Fisher Scientific. Hydrolysis enzymes exonuclease III (200 U/μL) and exonuclease I (20 U/μL) were purchased from Thermo Fisher Scientific. T4 NMP (nucleotide monophosphate) kinase was purchased from Jena Bioscience. *E. coli* S30 Extract System (with luciferase plasmid DNA template) was purchased from Promega Corporation.

##### DNA Template and Primers for PCR

Plasmid luciferase DNA (pBEST luc, 4864 bp) was from the *E. coli* S30 extraction kit. Plasmid DNA template encoding the SARS-CoV-2 envelope sequence (2019-nCoV_E Positive) was purchased from Integrated DNA Technologies company. Primers were designed from published sequences^19^ without 5′ *Nde*I and 3′ *Bam*HI restriction site sequences. The amplification length is 1653 base pairs (16–1668) of the luc sequence. Primers (oligonucleotides) with or without the 5′-terminal Fam modification, and with or without the 3′-phosphorothiolate (PS) modification, were purchased from Biomers Gmbh, Germany. The 3′-terminal PS tail was labeled by “*” in the flowing sequences (forward primer (Fam): 5′-atg gaa gac gcc aaa aac at*a *a*a*g-3′; reverse primer (Fam): 5′-tta caa ttt gga ctt tcc gc*c *c*t*t-3′).

##### Primers for Quantitative PCR (qPCR)

The DNA template for qPCR is the same as that for PCR (luc template). Primers were designed by IDT PrimerQuest Tool, with the forward primer starting from 673 of the luciferase sequence, the reverse primer starting from 805 of the luciferase sequence, and the length of amplicon being 133 base pairs (the same template and primer sequences as those for qPCR in the DNA NaCRe system^12^). Primers were purchased from Biomers Gmbh. The 3′-terminal PS tail was labeled by “*” in the flowing sequences (forward primer (Fam): 5′-cgc atg cca gag atc cta *t*t-3′; reverse primer (Fam): 5′-aga cga ctc gaa atc cac ata *t*c-3′). Primers with a sugar modification for the amplification of 2019-nCoV_E Positive plasmid DNA by qPCR (forward primer: 5′-cag cca ctg gta aca gga ttA g-3′; reverse primer: 5′-gca gag cgc aga tac caa aTa -3′) were purchased from Biomers, Gmbh. The modifications, 2′-Lock (2′-locked nucleic acid), 2′-OMe (2′-*O*-methyl), 2′-MOE (2′-*O*-methoxyethyl), and 2′-F (2′-deoxy-2′-fluoro), are at the penultimate position (labeled with a capital letter in the sequences). The backbone 2PS modification is labeled with *, between the last three nucleobases (forward primer: 5′-cag cca ctg gta aca gga tt*a *g-3′; reverse primer: 5′-gca gag cgc aga tac caa a*t*a-3′).

### Instruments

The Eppendorf ThermoMixer (RTM F1.5, 220–240 V/50–60 Hz) was purchased from Eppendorf. The horizontal gel electrophoresis system was purchased from Bio-Rad. Gel images were obtained with a GelDoc Go system, Bio-Rad. PCR was performed using a Proflex 3 × 32-well PCR thermal cycler system (Thermo Fisher Scientific). qPCR was performed using a QuantStudio 7 qPCR instrument (Applied Biosystems). HPLC was performed using an Infinite 1260 HPLC with C 18 column, Agilent.

#### Thermocycling Conditions for PCR

Initial denaturing (95 °C, 2 min) for one cycle, amplification (denaturing at 95 °C for 30 s, annealing at 55 °C for 30 s, and extension at 72 °C for 1 min) for 35 cycles, final extension (72 °C for 10 min) for one cycle, and cooling to 4 °C. The PCR amplification products were used for the recycling of dNTPs, regeneration of the PCR kit, and recycling of primers. Unless specified, all PCRs were processed under the same thermocycling conditions.

#### Gel Electrophoresis

The agarose gel was run in 1× TAE buffer at 120 V for 40 min. Afterward, the gel was stained by 1× Sybr safe solution for 40 min under slow shaking. Thereafter, the image of the gel was obtained by a GelDoc Go under Sybr safe channel with 1 s exposure time. Unless specified, all agarose gel experiments were processed under the same conditions.

#### Thermocycling Conditions for qPCR

Initial denaturing (95 °C, 2 min) for one cycle, amplification (denaturing at 95 °C for 30s, annealing at 55 °C for 30 s, and extension at 72 °C for 1 min) for 35 cycles, final extension (72 °C for 10 min) for one cycle, and cooling to 4 °C. The PCR amplification products were used for the recycling of dNTPs, regeneration of the PCR kit, and recycling of primers. Unless specified, all PCRs were processed under the same thermocycling conditions.

#### MALDI-TOF

All MALDI-TOF spectra were collected using a Bruker AutoFlex Speed instrument (Bremen, Germany).

#### Oligo-Pro II Capillary Electrophoresis

The Agilent Oligo Pro II system with DN-415 OLIGEL ssDNA Gel was used in the corresponding methods.

### Recycling of dNTPs from PCR Products/Waste

#### Nuclease Resistance Evaluation of 4PS-Modified Primers

##### Nuclease-Resistance Test of PS-Modified Primers

The nuclease-resistance capacity of PS-modified primers was tested by mixing Fam-FP-PS, Fam-RP-PS, Fam-FP, and Fam-RP (0.5 μL, 100 μM) with exonuclease III (5 μL, 500 Units) and exonuclease I (5 μL, 50 Units); 10 μL of 10× exonuclease III buffer, 79.5 μL of nuclease-free water (total 100 μL), and the nuclease hydrolysis mixtures were incubated at 37 °C and 350 rpm for 2, 6, and 24 h, respectively. Further, the hydrolysis reaction mixtures (5 μL each) were loaded onto 2% agarose gel. As shown in the gel (Figure S1b), the FAM dye-labeled forward and reverse primers with PS protection (Fam-FP-PS and Fam-RP-PS) can tolerate enzymatic hydrolysis for 24 h (lanes 1–3 (Fam-FP-PS) and lanes 7–9 (Fam-RP-PS)). In contrast, the forward and reverse primers without a PS tail (Fam-FP and Fam-RP) were hydrolyzed with very less residues after 2 h (lane 6 (Fam-FP) and lane 9 (Fam-RP)). This shows that the primers with a 4PS tail modification can resist nuclease-catalyzed hydrolysis.

#### PCR Amplification and Recycling of dNTPs

##### Preparation of the PCR Reaction Mixture

DreamTaq master mix (2×, 50 μL), forward primer (0.5 μL, 100 μM), reverse primer (0.5 μL, 100 μM), Luc DNA template (0.5 μL, 100 ng/μL), and nuclease-free water (48.5 μL) were mixed in a total of 100 μL. The PCR products (triplicate, primers with or without 4PS modification) were extracted using a PCR extraction kit, and the concentration of PCR products was quantified by Nanodrop.

##### Cleavage and Hydrolysis

The PCR product was first heated to 100 °C and incubated for 15 min to deactivate polymerase. Further, 50 μL of the PCR product was mixed with 3.25 μL of Dedl (32.5 Units), 4 μL of Exo III, and 4 μL of Exo I and incubated at 37 °C and 350 rpm overnight. At the cleavage site of Dedl (C∧TNAG; 629 and 1043 of the luc sequence), 5′-terminal overhangs with a length of four nucleotides were generated, which are suitable for the binding of Exo III to the cleaved PCR product and initiation of hydrolysis. Afterward, the hydrolysis mixture was heated to 80° and incubated for 15 min to inactivate the restriction and hydrolysis enzymes. As a result of the increase in volume by adding the cleavage and hydrolysis enzymes, there was a 1.225× dilution of the reaction mixture.

##### Phosphorylation

The PCR product hydrolysis mixture (20 μL) was mixed with 1.2 μL of *E. coli* S30 extract (20× dilution), 1 μL of T4 dNMP kinase (50× dilution, 2 Units), 1 μL of ATP (3 mM), and 0.77 μL of acetylphosphate lithium potassium (AceP, 50 mM), in a total of 24 μL. The phosphorylation reaction mixture with a final volume of 24 μL, with estimated amounts of dNMPs (on average, about 0.15 mM each), ATP (0.125 mM), and AceP (1.6 mM, 1.5 equiv), was incubated in a thermomixer at 400 rpm at 37 °C for 4 h. Afterward, all hydrolysis and phosphorylation enzymes and nonhydrolyzed DNA were removed by ultrafiltration (Amicon, 3 kD cutoff, 5000 rpm for 10 min at 4 °C). As a result of the increase in volume by adding the phosphorylation reagents, there was a 1.2× dilution of the reaction mixture.

#### Quantification of Recycled dNTPs by HPLC

The concentration of recycled dNTPs was determined by HPLC with a C-18 reverse-phase column. Mobile-phase Buffer A: 5 mM *t*-butyl ammonium phosphate, 10 mM KH_2_PO_4_, and 0.25% methanol adjusted to pH 6.9. Buffer B: 5 mM *t*-butyl ammonium phosphate, 50 mM KH_2_PO_4_, and 30% methanol (pH 7.0). From 0 to 15 min, the gradients of buffer A/B changed from 40/60 to 20/80%, were run under the same gradient conditions until 20 min, and then changed back to the starting condition of 40/60%, with a flow rate of 0.5 mL/min.

##### HPLC Quantification

Further, the filtered reaction mixture (dNTPs_post PCR, dNTPs_post hydrolysis, and dNTPs_post phosphorylation) was diluted (50×) and injected into the HPLC column (50 μL) for the quantification of dNTPs. The mixture of dNMPs (2.5 μM each) and dNTPs from the original PCR kit (100× dilution, 4 μM each) was injected into the HPLC column (50 μL) as a positive control. For retention times of the above five samples, see Figure S2a. The calibration curve was achieved by serial dilution of dNTPs and ATP (2.5–40 μM, Figure S2b). The final concentration of dNTPs was multiplied with the dilution factor during the PCR kit regeneration process (1.225× post hydrolysis and 1.2× for phosphorylation). After PCR amplification, about 70% of the dNTPs was consumed (Figure S2c,d). In addition, new peaks were generated (Figure S2a_dNTPs_post PCR), which were attributed to hydrolysis products of dNTPs (dNMPs and dNDPs) probably induced by the heating under PCR conditions. After enzymatic hydrolysis, the amount of dNMPs was highly increased (Figure S2a: post hydrolysis; retention time of additional fractions between 3 and 14 min). There is a slight decrease in the dNTP residue, which might be caused by enzymatic hydrolysis. After phosphorylation, the dNMP peaks almost disappeared, and the dNTP peaks were regenerated, showing excellent phosphorylation efficiency (Figure S2a_post phosphorylation). The recycling efficiency of dNTPs is 74.49 ± 2.09%, and the final concentration of all dNTPs is 101.35 ± 2.84 μM (Figure S2c,d).

### Recycling of the PCR Kit

#### PCR Amplification and Regeneration of PCR Substrates

##### PCR Amplification of luc DNA

The ratio of DNA template and primers used for PCR amplification was the same as that mentioned above. Although a relatively good monomer recycling efficiency is achieved, we cannot confirm that all PCR residues have been removed and no contaminants will be carried over to the next round of PCR. To improve the hydrolytic efficiency, we further split the hydrolysis of PCR product into two steps with the restriction enzymes added only in the second step.

##### Cleavage and Hydrolysis

The PCR product was first heated to 100 °C and incubated for 15 min to deactivate polymerase. Further, 50 μL of the PCR product was mixed with 1.5 μL of Exo III and 1.5 μL of Exo I and incubated at 37 °C and 350 rpm overnight. Afterward, the hydrolysis mixture was heated to 80 °C and incubated for 15 min to inactivate the hydrolysis enzymes. Next, 1 μL of DedI (10 Units), 1.5 μL of Exo III, and 1.5 μL of Exo I were added to the hydrolysis mixture and incubated at 37 °C and 350 rpm for 8 h. Afterward, the cleavage and hydrolysis mixture was heated to 80 °C and incubated for 15 min to inactivate the restriction and hydrolysis enzymes. As a result of the increase in volume by the two steps of hydrolysis and cleavage, there was a 1.12× (50–56 μL) dilution of the reaction mixture.

##### Phosphorylation

The phosphorylation step was processed by a method similar to that mentioned above. As the phosphorylation mixture would increase the total volume of the reaction mixture and decrease the concentration of all reagents in the PCR substrate, we would like to minimize the amount of added phosphorylation mixture. Therefore, we first prepared a phosphorylation reagent mixture (10 μL) with T4 60× dilution, S30 20× dilution, ATP 2.46 mM, and AceP 31.5 mM. The PCR hydrolysis mixture (30 μL) was mixed with 2 μL of the prepared phosphorylation reagents, and the phosphorylation mixture was incubated in a thermomixer at 400 rpm at 37 °C for 4 h. Afterward, the reaction mixture was directly subjected to PCR amplification. As a result of the increase in volume by adding the phosphorylation reagents, there was a 1.066× dilution (30–32 μL) of the reaction mixture.

From the recycling efficiency of monomers as well as the dilution factor for each step of PCR kit regeneration, the final concentration of dNTPs in the regenerated PCR kit is calculated as follows:
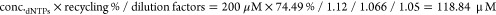


#### Recycling of the Regenerated PCR Substrate

##### Preparation of the PCR Reaction Mixture

The regenerated PCR substrate (20 μL), 0.5 μL of luc DNA template (20 ng/μL), and 0.5 μL of DreamTaq polymerase (diluted to 1 U/μL) were mixed in a total volume of 21 μL.

Preparation of the No-DNA Template Control

The regenerated PCR substrate (20 μL), 0.5 μL of nuclease-free water, and 0.5 μL of DreamTaq polymerase (diluted to 1 U/μL) were mixed in a total volume of 21 μL. As a result of the increase in volume by adding the DNA template and the polymerase, there was a 1.05× dilution (21–20 μL) of the reaction mixture. The whole process for PCR amplification, regeneration, and second round of PCR was processed by primers without PS modification as a negative control.

##### Gel Electrophoresis

After the PCR thermocycles, the first and second rounds of the PCR product as well as the PCR hydrolysis mixture were loaded onto 2% agarose gel. All samples were mixed with 2 μL of 6× DNA loading dye. Lane 1, TrackIt 100 bp ladder, 2 μL; lane 2, first round of PCR product, 8.4 + 1.6 μL water; lane 3, PCR hydrolysis mixture, 9.375 + 0.625 μL water; lane 4, second round of PCR product with DNA template added, 10 μL; lane 5, second round of PCR product without DNA template added, 10 μL. Lanes 6–8, first and second rounds of PCR products (primers without PS modification); lane 6, first round of PCR product, 8.4 + 1.6 μL water; lane 7, PCR hydrolysis mixture, 9.375 + 0.625 μL water; lane 8, second round of PCR product with DNA template added, 10 μL.

#### Recycling of Primers

##### PCR Amplification of luc DNA

The ratio of DNA template and primers used for PCR amplification (duplicate) was the same as that mentioned above. The concentration of primers was determined by Nanodrop (ssDNA method, 1433.05 ng/μL at 100 μM), and the amount of added primers was 716.5 ng (0.5 μL, 100 μM).

##### Cleavage and Hydrolysis

The PCR product (100 μL) was first heated to 100 °C and incubated for 15 min to deactivate polymerase. Further, the PCR product was mixed with BshNI (*Ban*I) (2.5 μL, 25 Units). At the cleavage site of BshNI (*Ban*I) (G∧GYRCC, 48 of the luc sequence), 5′-terminal overhangs with a length of four nucleotides were generated. Another restriction enzyme PsuI (*Bst*YI) (10 μL, 100 Units) with multiple cleavage sites (R∧GATCY, 613, 684, 1137, and 1625 of the luc sequence) was also added to cleave the PCR product. Also, the cleaved fragment with the forward primer was shortened to 33 nt (16–48), and the cleaved fragment with a reverse primer was shortened to 43 nt (1625–1668) for better hydrolysis and recycling efficiency. The cleavage mixture was incubated at 37 °C and 350 rpm for 5 h. Afterward, the hydrolysis mixture was heated to 80 °C and incubated for 20 min to inactivate the cleavage enzymes. Next, 15 μL of Exo III and 15 μL of Exo I were added to the hydrolysis mixture and incubated at 37 °C and 350 rpm for 8 h. Afterward, the hydrolysis mixture was heated to 80 °C and incubated for 15 min to inactivate the hydrolysis enzymes. Further, the PCR cleavage–hydrolysis mixture was concentrated by ultrafiltration (3 K cutoff, 8000 rpm, 45 min, 4 °C) to 50 μL. The mixture of primers was extracted using a “Oligo Clean-Up and Concentration Kit.” The concentration of extracted primers was quantified by Nanodrop (ssDNA method) with a final amount of 436 ± 7.2 ng (40 μL, 10.90 ± 0.18 ng/μL).

##### Recycling Yield (Mass Ratio)

436 ± 7.2 ng/716.5 ng = 60.85 ± 0.01%.

##### MALDI-TOF

The molecular masses of fresh primers and recycled primers were characterized by MALDI-TOF. The matrix was prepared as follows: a 90:10 mixture of (1) 50 mg/mL 3-hydroxy picolinic acid in 1:1 water/acetonitrile and (2) 100 mg/mL diammonium hydrogen citrate in water.^19^ All of the MALDI-TOF spectra were collected using a Bruker AutoFlex Speed instrument (Bremen, Germany). The forward primer (2.5 μM in water), reverse primer (2.5 μM in water), and recycled primers (10.90 ± 0.18 ng/μL in water) were mixed with an equal volume of the matrix solution. For each sample, a 1 μL aliquot of such a solution mixture was deposited and dried onto a stainless ground steel target plate. Measurements were performed in positive ionization mode and operated in the linear mode in the 1–14 K *m*/*z* mass range. The laser intensity was kept at around 80% for all measurements. Typically, around 1000 shots were accumulated for each spectrum. Mass spectra were processed with Flex Analysis (Bruker) software.

##### Recycling Yield (Molar Ratio)

Because of the shortened length of primers, the molecular weight of recycled primers was decreased from 7.5 and 7.3 to 6.9 kD (on average). Therefore, the recycling yield calculated by the molar ratio is 64.38 ± 0.01%. The concentration of recycled primers thus calculated from the molar ratio was 0.82 μM.

##### Preparation of the PCR Reaction Mixture

(1) Positive control of PCR with fresh primers: DreamTaq master mix (2×, 10 μL), a mixture of forward and reverse primers (8 μL, 1.25 μM), Luc DNA template (1 μL, 10 ng/μL), and nuclease-free water (1 μL) were mixed in a total volume of 20 μL. (2) PCR with recycled primers: DreamTaq master mix (2×, 10 μL), recycled primer mixture (8 μL, ∼0.82 μM), Luc DNA template (1 μL, 10 ng/μL), and nuclease-free water (1 μL) were mixed in a total volume of 20 μL. The thermocycler conditions are the same as the above settings. After the PCR amplification, PCR products from fresh and recycled primers (10 μL each) were loaded onto 2% agarose gel.

### Recycling Primers for qPCR

#### Nuclease Resistance Evaluation of 4PS-Modified Primers

##### Nuclease-Resistance Test of PS-Modified Primers

The nuclease-resistance capacity of PS-modified primers was tested by mixing 0.5 μL (100 μM) of Fam-FP-PS, Fam-RP-PS, Fam-FP, or Fam-RP with exonuclease III (5 μL, 500 Units) and exonuclease I (5 μL, 50 Units), 10 μL of 10× exonuclease III buffer, and 79.5 μL of nuclease-free water (total 100 μL) and incubating overnight at 37 °C and 350 rpm. Further, the hydrolysis reaction mixtures (5 μL each) were loaded onto 2% agarose gel. As shown in the gel (Figure S1), the FAM dye-labeled forward and reverse primers with PS protection (Fam-FP-PS and Fam-RP-PS) can tolerate enzymatic hydrolysis (lanes 2 and 4). There is no obvious difference in the bands in comparison with the control samples of nuclease-free primers (lanes 1 and 3). In contrast, the forward and reverse primers without a PS tail (Fam-FP and Fam-RP) were hydrolyzed with very few residues (lanes 6 and 8) in comparison with the control samples of nuclease-free primers (lanes 5 and 8). This shows that the PS tail modification of primers could resist nuclease-catalyzed hydrolysis.

As qPCR experiments are normally processed on a very small scale (10 or 20 μL for each sample), next, we tried to recover primers from PCR product/waste (100 μL scale for qPCR).

##### Preparation of the PCR Reaction Mixture

PowerTrack SYBR Green Master Mix (2×, 50 μL), a mixture of forward and reverse primers (1.6 μL, 25 μM) with a final concentration of 400 nM, Luc DNA template (1 μL, 1 ng/μL), and nuclease-free water (47.4 μL) were mixed in a total volume of 100 μL (duplicate, with 4 × 100 μL in each group). The concentration of primers was quantified by Nanodrop (ssDNA method, 361.12 ng/μL at 25 μM), and the amount of added primers was 577.79 ng/100 μL of PCR mixture (1.6 μL).

##### Thermocycling Conditions for PCR

Initial denaturing (95 °C, 2 min) for one cycle, amplification (denaturing at 95 °C for 5 s, annealing, and amplification at 60 °C for 15 s) for 35 cycles, and final extension (72 °C for 10 min) for one cycle and cooling to 4 °C.

##### Cleavage and Hydrolysis

The PCR product was first heated to 100 °C and incubated for 15 min to deactivate polymerase. Further, 100 μL of the PCR product was mixed with Kpn2I (*Bsp*EI) (1.5 μL, 15 Units). At the cleavage site of Kpn2I (*Bsp*EI) (T∧CCGGA, 711 of luc sequence), 5′-terminal overhangs with a length of four nucleotides were generated. The amplified DNA was cleaved into two shorter fragments (Figure S7a, lengths of 39 and 94 nt, respectively). The DNA cleavage mixture was incubated at 55 °C and 350 rpm overnight. Afterward, the reaction mixture was heated to 80 °C and incubated for 20 min to inactivate the cleavage enzymes. Next, 5 μL of Exo III and 5 μL of Exo I were added to the hydrolysis mixture and incubated at 37 °C and 350 rpm for 1.5 h. Afterward, the hydrolysis mixture was heated to 80 °C and incubated for 15 min to inactivate the hydrolysis enzymes. Further, the PCR cleavage–hydrolysis mixture (all 4 × 100 μL) was concentrated by ultrafiltration (3 K cutoff, 8000 rpm, 45 min, 4 °C) to 50 μL. The mixture of primers recycled from the quadruplicates was extracted using an “Oligo Clean-Up and Concentration Kit.” The concentration of extracted primers was quantified by Nanodrop (ssDNA method) with a final amount of 873.2 ± 10.4 ng (40 μL, 21.83 ± 0.26 ng/μL, duplicate). The molecular masses of fresh primers and recycled primers were characterized by MALDI-TOF by the same method as above.

##### Recycling Yield (Molar Ratio)

As a result of the shortened length of primers, the molecular weight of recycled primers was decreased from 6.1 and 7 to 6.4 KD (on average, multiplied by 0.976). Therefore, the recycling yield calculated by the molar ratio is 873.2 ± 10.4 ng/(577.79 × 4)/(150/160)/0.976 = 40.93 ± 0.48%. As a result of the loss during oligo extraction (up to 90% recovery rate with at least 10% loss), the preserved primers should be more than 50%. The as-obtained recycled primers were further diluted to a stock solution (1.33 μM).

#### Monitoring of the Primer Recycling by Oligo-Pro II Capillary Electrophoresis

##### Sample Preparation and Methods

All samples were diluted to 0.25 μM by adding 5.0 μL of 1.0 μM stock to 15.0 μL of nuclease-free water with mineral oil overlay. All samples were analyzed on the Agilent Oligo Pro II system with DN-415 OLIGEL ssDNA Gel using the corresponding methods.

##### Sample Injection

Mixture of fresh primers: 7 kV for 5 s; sample separation: 12 kV for 90 min. Sample injection for the mixture of recycled primers: 10 kV for 15 s; sample separation: 12 kV for 90 min.

##### Data Analysis

The Agilent Oligo Pro II data analysis software with the following integration parameters (Table 1) was used for automated data processing. The parameters were adjusted as needed to optimize the integration of individual samples in each run. The percentage purities of all integrated peaks are 87.94% (fresh primers), 48.14% (recycled primers, 1 h hydrolysis), 85.07% (recycled primers, 2 h hydrolysis), and 89.29% (recycled primers, 4 h hydrolysis).

**Table 1:** Oligo Pro II Automated Data Processing Setting

data analysis parameters initial settings	0.25 μM samples 1–20
peak analysis	
peak detection window (s)	3
minimum peak height	1
inclusion region	
start time (min)	25
end time (min)	65
manual baseline	
start time (min)	25
end time (min)	65

##### Preparation of Duplicate qPCR Mixtures

(1) Fresh primers for qPCR: PowerTrack SYBR Green Master Mix (2×, 5 μL), a fresh mixture of primers (2.5 μM, 1.6 μL), Luc DNA template, 1 μL of serial dilutions of 1, 0.1, 0.01, and 0.001 ng/μL, 1 μL of nuclease-free water as the no-template control (NTC) sample, and 3.4 μL of nuclease-free water were mixed in a total volume of 10 μL. In the final qPCR mixture, the concentration of primers is 400 nM. (2) Recycled primers for qPCR: PowerTrack SYBR Green Master Mix (2×, 5 μL), recycled primers, (1.33 μM, 3 μL), Luc DNA template, 1 μL of serial dilutions of 1, 0.1, 0.01, and 0.001 ng/μL, and 1 μL of nuclease-free water as the NTC were mixed in a total volume of 10 μL. In the final qPCR mixture, the concentration of primers is 400 nM.

##### Thermocycling Conditions for qPCR

The qPCR amplification was performed by a QuantStudio 7 qPCR system: initial denaturing (95 °C, 2 min) for one cycle and amplification (denaturing at 95 °C for 15 s, annealing, and amplification at 60 °C for 30 s) for 40 cycles.

#### Nuclease Tolerance of Ribose Sugar-Modified Primers

##### Step (1) Nuclease Hydrolysis

Six groups of primer mixtures (four with sugar modification, one with PS modification, and one without modification) are prepared. The hydrolysis mixture (primers 100 μM, 2.5 μL; Exo I 31.25 μL; 10x Exo I buffer 10 μL; nuclease-free water 56.25 μL; total 100 μL) is prepared. The mixture is incubated at 37 °C and 350 rpm for 1.5 or 4 h. Afterward, the temperature is increased to 80 °C and incubated for 15 min to inactivate the hydrolysis enzymes. Fast centrifugation is carried out to cool down the reaction mixture. The reaction mixture is stored for qPCR and Maldi experiments.

##### Step (2) qPCR

The qPCR mixture was prepared in triplicate as follows: PowerTrack SYBR Green Master Mix (2×, 5 μL) and a mixture of six groups of fresh NaCRe primers (2.5 μM, 1.6 μL) were combined. In the final qPCR mixture, with the concentration of primers of 400 nM, DNA template, 1 μL of serial 10× dilutions of 200,000, 20,000, and 2000 copies or 1 μL of nuclease-free water as the no-template control (NTC) sample, and 2.4 μL of nuclease-free water are added in a total of 10 μL.

The qPCR mixture using primers with nuclease treatment was prepared in triplicate as follows: PowerTrack SYBR Green Master Mix (2×, 5 μL), a mixture of six groups of NaCRe primers after 1.5 or 4 h of nuclease treatment were mixed (2.5 μM, 1.6 μL). In the final qPCR mixture, (the concentration of primers is 400 nM; quantification generated by the concentration of fresh primers), DNA template, 1 μL of serial 10× dilutions of 200,000, 20,000, and 2000 copies or 1 μL of nuclease-free water as the no-template control (NTC) sample, and 2.4 μL of nuclease-free water were added in a total of 10 μL.

##### Step (3) Thermocycling Conditions for qPCR

The qPCR amplification was performed by a QuantStudio 7 qPCR system with initial denaturing (95 °C, 2 min) for one cycle and amplification (denaturing at 95 °C for 15 s, annealing, and amplification at 60 °C for 60 s) for 40 cycles.

## Supporting Information Available

Additional HPLC chromatograms, mass spectra, and images of agarose gel (PDF)
