# Supplementary material for: Recycling of Polymerase Chain Reaction (PCR) Kits
Source: ACS Sustain Chem Eng. 2023 Mar 24;11(14):5524–36. doi: 10.1021/acssuschemeng.2c07309 (PMC10081572; doi:10.1021/acssuschemeng.2c07309)
Supplement: Supplementary file 1 — sc2c07309_si_001.pdf [file sc2c07309_si_001.pdf]

Supporting information for

Recycling of Polymerase Chain Reaction (PCR) Kits

Weina Liu,[†,‡,§]* *Yong Zhu,*[†,‡,§] *Francesco Stellacci*[†,‡]*

†Institute of Materials, École Polytechnique Fédérale de Lausanne, Switzerland. Station 12, Lausanne 1015, Switzerland

‡Institute of Bioengineering, École Polytechnique Fédérale de Lausanne, Switzerland. Station 12, Lausanne 1015, Switzerland.

§W.L. and Y.Z. contributed equally to this paper

*Correspondence: weina.liu@epfl.ch

francesco.stellacci@epfl.ch

Number of pages: 8

Number of Figures: 8

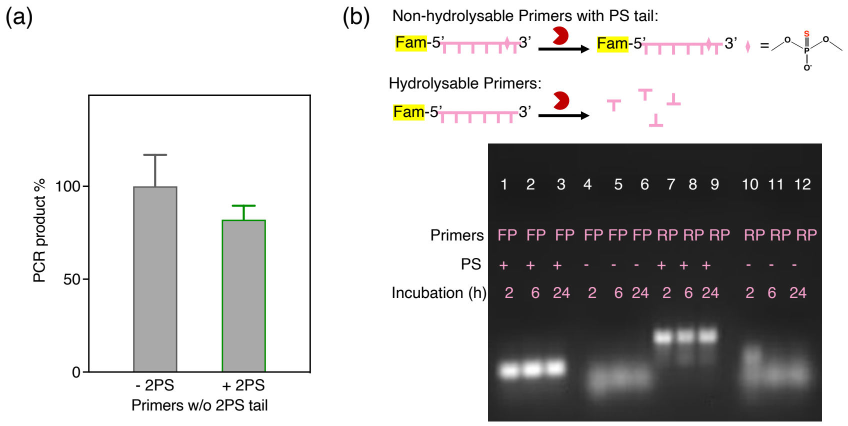

Figure S1. (a) Yield of PCR products by using primers with or without 4PS-modification. (b) Nuclease tolerance study of primers with or without 4PS-modification. Lane 1-3, forward primer with 4PS-modification incubated with mixture of nucelases for 2, 6 and 24h. Lane 4-6, forward primer without 4PS-modification incubated with mixture of nucelases for 2, 6 and 24h. Lane 7-9, reverse primer with 4PS-modification incubated with mixture of nucelases for 2, 6 and 24h. Lane 10-12, reverse primer without 4PS-modification incubated with mixture of nucelases for 2, 6 and 24h.

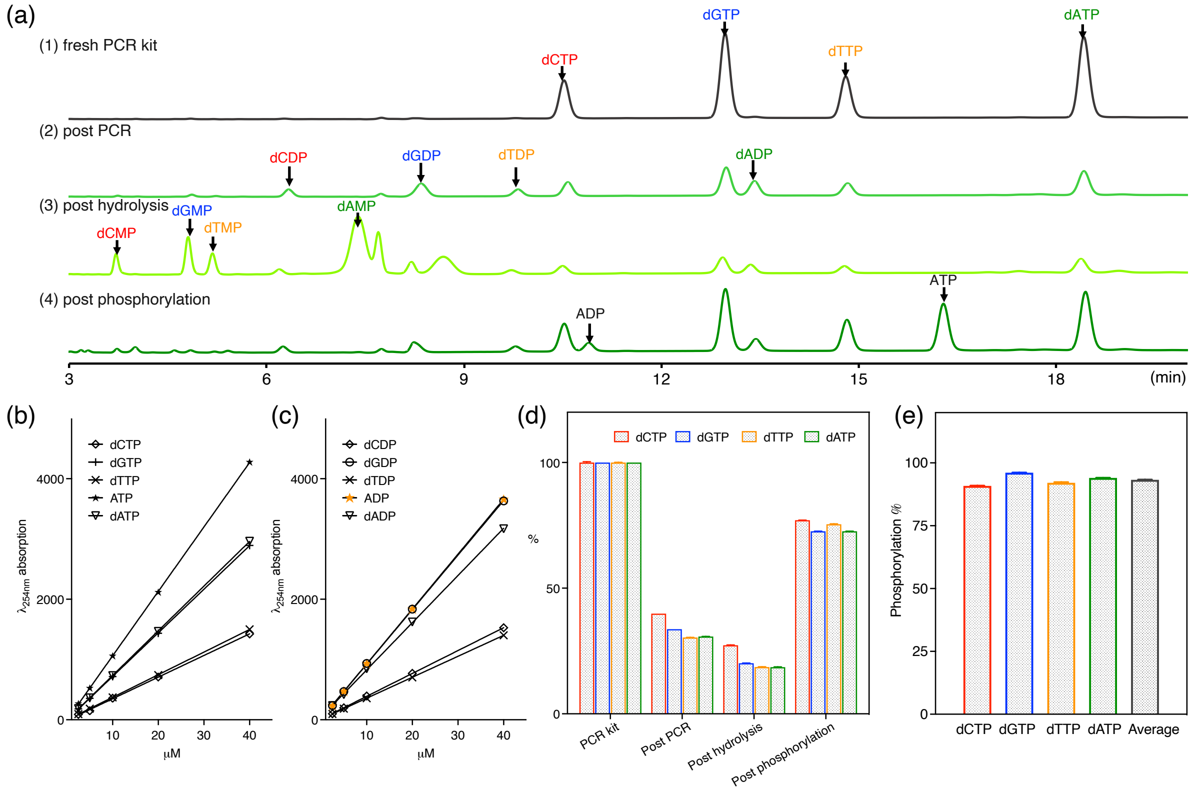

Figure S2. (a) HPLC retention time of monomeric nucleotides for each step of PCR kit regeneration (fresh PCR kits with dNTPs standards (line 1), dNTPs residues after PCR (line 2, post PCR), PCR product hydrolysis mixture (line 3, post hydrolysis), and regenerated PCR substrate (line 4, post phosphorylation). (b) Calibration curve of dNTPs and ATP with concentration 2.5, 5, 10, 20, 40 µM for each. (c) Calibration curve of dNDPs and ADP with concentration 2.5, 5, 10, 20, 40 µM for each. (d) Residue of dNTPs at each step of PCR kit regernation. After PCR, dNTPs were consumed and the amount of dNTPs was decreased to dC 39.86 ± 0.01%, dG 33.69 ± 0.01%, dT 30.36 ± 0.06%, dA 30.77 ± 0.03%, in average 33.67 ± 4.06%. After enzymetic hydrolysis dNTPs were partialy hydrolyzed, and the amount of dNTPs residues was decreased to dC 27.29 ± 0.11%, dG 20.15 ± 0.04%, dT 18.62 ± 0.10%, dA 18.57 ± 0.10%, in average 21.16 ± 3.84%. After phosphorylation, the dNMPs were converted to dNTPs, and the amount of dNTPs was increased to dC 77.04 ± 0.05, dG 72.64 ± 0.02%, dT 75.42 ± 0.19%, dA 72.65 ± 0.04%, in average 74.44% ± 2.01%. (e) Phosphorylation effieicency of dC 90.85 ± 0.06%, dG 96.11 ± 0.03%, dT 92.09 ± 0.23%, dA 94.00 ± 0.05%, in average 93.26 ± 2.12%.

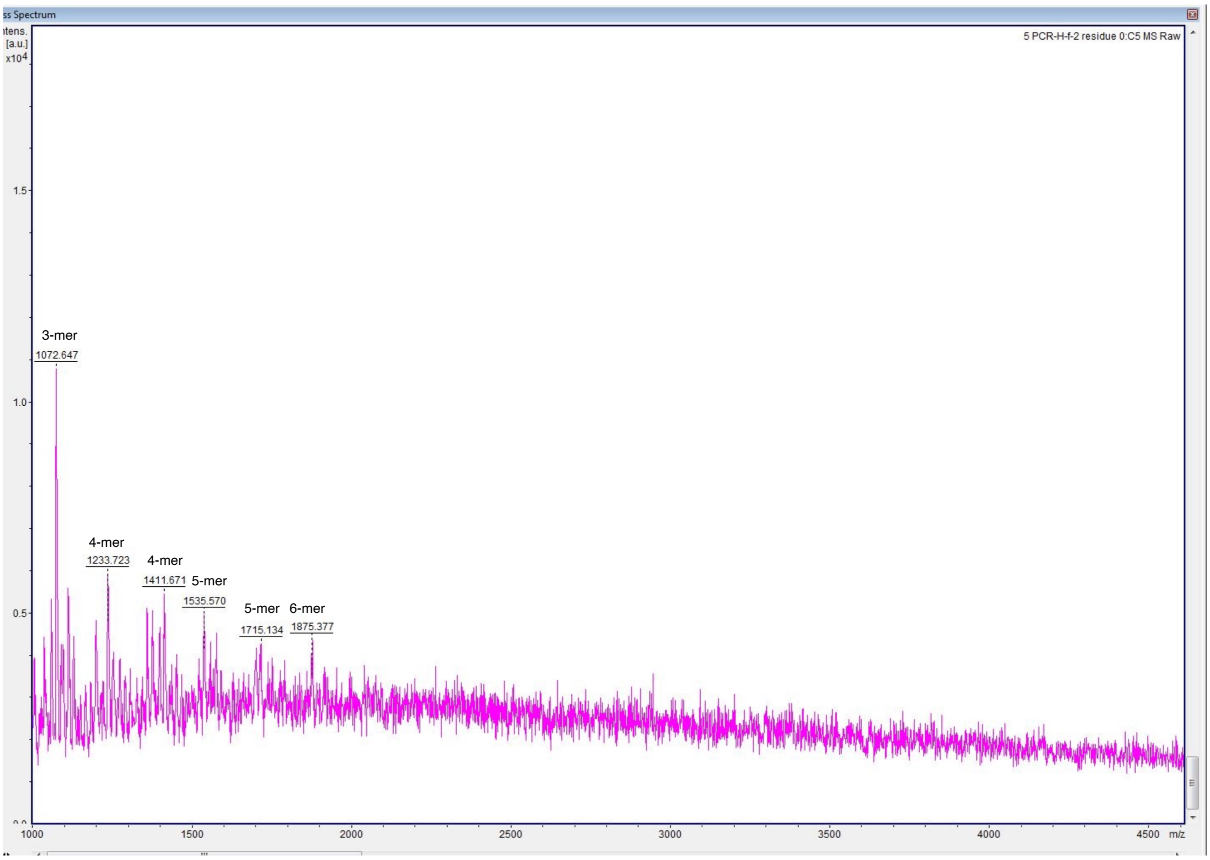

Figure S3. Mass spectrum of oligonucleotite residues with length of 3-6mer in the regenerated PCR kit.

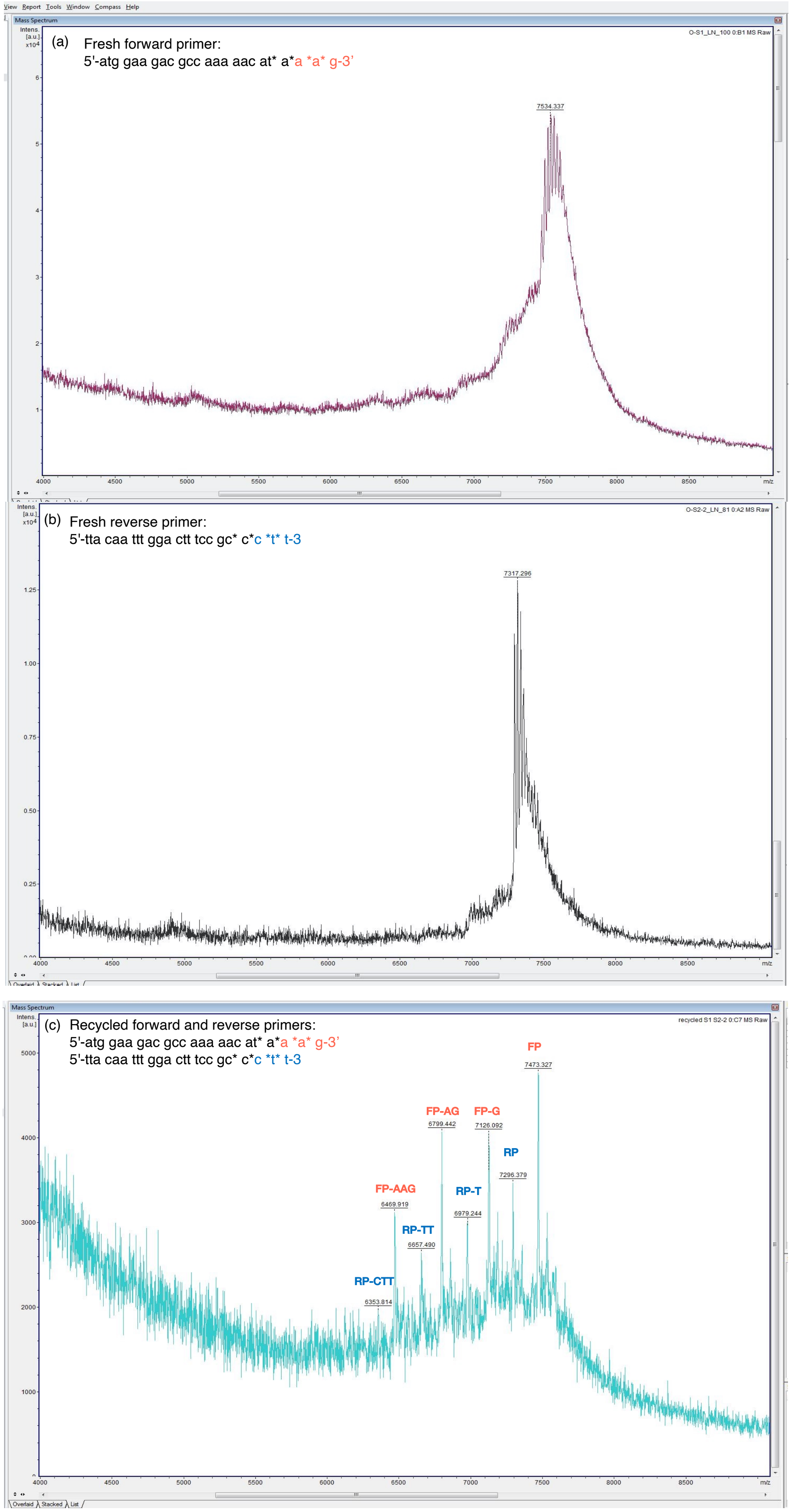

Figure S4. Mass spectrum of (a) 4PS-modified forward primer, (b) 4PS-modified reverse primers, and (c) recycled primers with 1-3 terminal nucleotide lost.

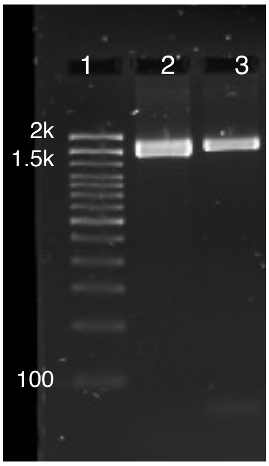

Figure S5. Agarose gel of PCR amplification products from fresh primers (lane 2, 400 nM of added primers) and recycled primers (lane 3, 263 nM of added primers).

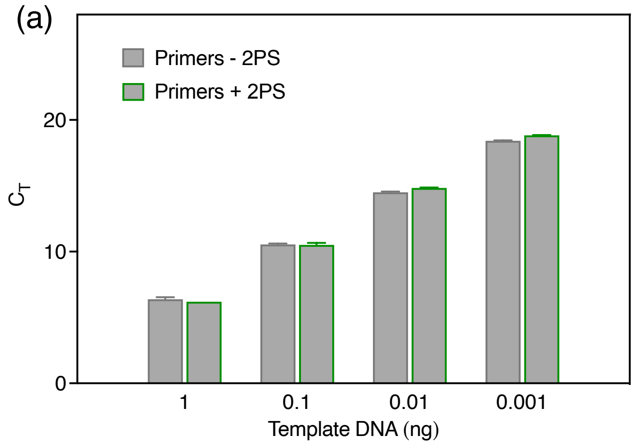

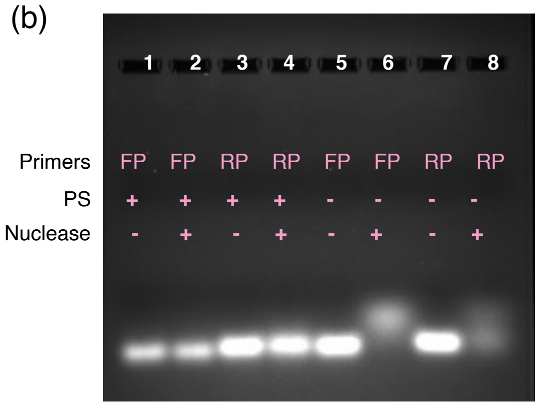

Figure S6. (a) qPCR performance by using primers with or without 2PS-modification with DNA template 1-0.001 ng. (b) nuclease resistance evaluation of primers with or without 2PS-modification. After overnight incubation with nuclease, 10 μL of each primer, or primer + nuclease mixtures were loaded to a 2% agarose gel, lane 1, FP-PS; lane 2, FP-PS + nuclease; lane 3, RP-PS; lane 4, RP-PS + nuclease; lane 5, FP; lane 6, FP + nuclease; lane 7, RP; lane 8, RP + nuclease.

(a) (b)

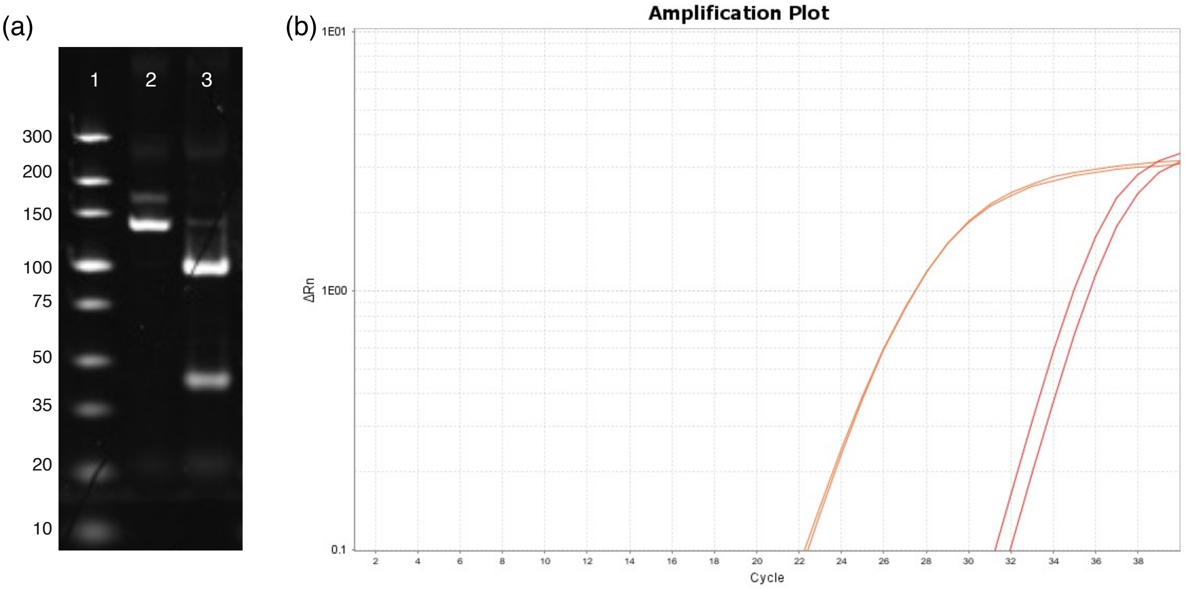

Figure S7. (a) Agarose gel of qPCR amplification product (lane 2) as well as the cleavage fragments (lane 3). (b) qPCR amplification plot of NTC (no template control) sample, red-qPCR kit prepared by recycled primers, orange-qPCR kit prepared by fresh primers.

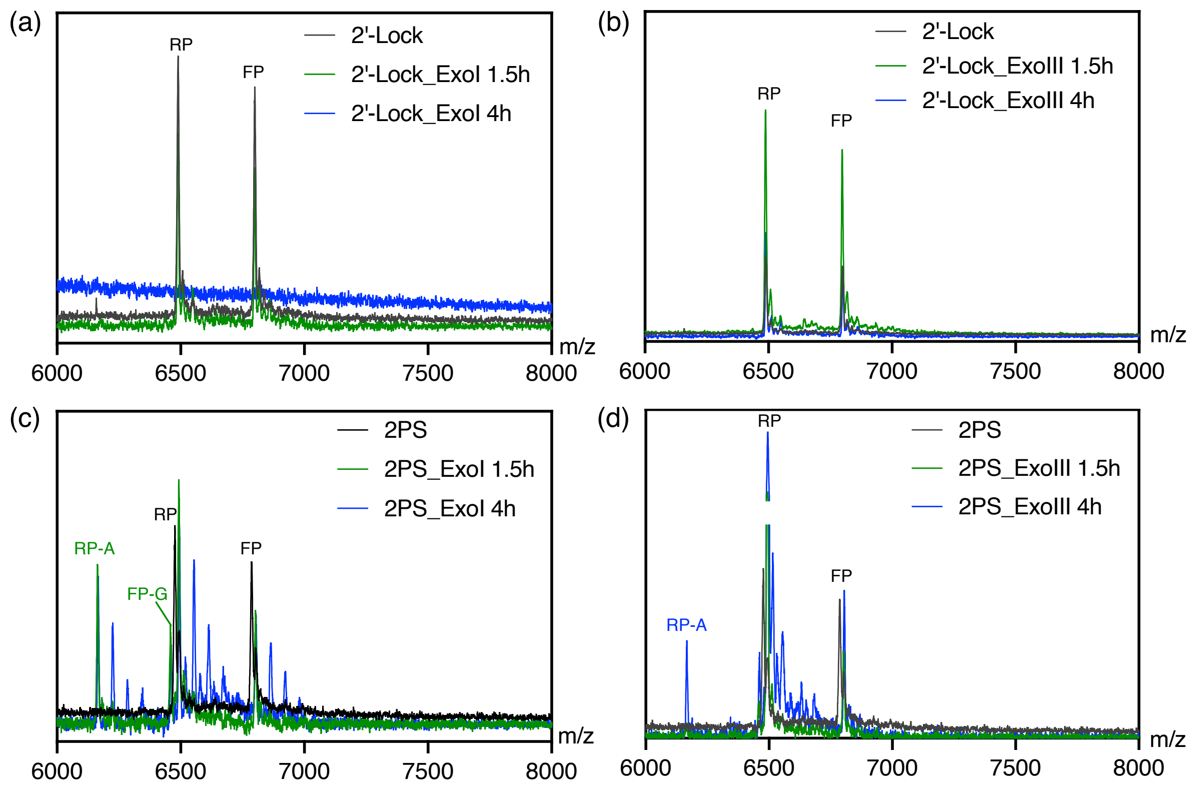

Figure S8. Maldi-Tof Mass spectra of qPCR primers with 2'-lock modification before and after 1.5h or 4h ExoI (a) and ExoIII (b) treatment, and mass spectra of qPCR primers with 2PS modification before and after 1.5h or 4h ExoI (c) and ExoIII (d) treatment. After 1.5h ExoI nuclease treatment, the molecular mass of 2'-lock modified primers remain the same, but after 4h nuclease treatment, the molecular mass of 2'-lock modified primers can not be detected anymore. After 1.5h and 4h ExoIII treatment, there was no obvious change of the molecular mass for 2'-lock modified primers. Those results showed that the 2'-lock modification can slowdown the nuclease ExoI hydrolysis, and totally resist the ExoIII hydrolysis. For 2PS modified primers, after 1.5h and 4h ExoI treatment, and 4h ExoIII treatment, molecular mass of primers with one terminal A or G lost were detected, showing that the 2PS can effectively slowdown the nuclease hydrolysis.
